# NOP10 predicts lung cancer prognosis and its associated small nucleolar RNAs drive proliferation and migration

**DOI:** 10.1038/s41388-020-01570-y

**Published:** 2020-12-07

**Authors:** Chunhong Cui, Yi Liu, Dennis Gerloff, Christian Rohde, Cornelius Pauli, Marcel Köhn, Danny Misiak, Thomas Oellerich, Schraga Schwartz, Lars-Henning Schmidt, Rainer Wiewrodt, Alessandro Marra, Ludger Hillejan, Frank Bartel, Claudia Wickenhauser, Stefan Hüttelmaier, Stefanie Göllner, Fengbiao Zhou, Bayram Edemir, Carsten Müller-Tidow

**Affiliations:** 1 Department of Medicine V, Hematology, Oncology and Rheumatology, University of Heidelberg, 69120 Heidelberg, Germany; 2 Department of Hematology and Oncology, Martin-Luther-University Halle-Wittenberg, 06120 Halle (Saale), Germany; 3 Institute of Molecular Medicine, Martin-Luther-University Halle-Wittenberg, 06120 Halle (Saale), Germany; 4 Department of Medicine II, Hematology/Oncology, Goethe University, 60590 Frankfurt, Germany; 5 German Cancer Consortium/German Cancer Research Center, 69120 Heidelberg, Germany; 6 Department of Molecular Diagnostics/Translational Proteomics, Frankfurt Cancer Institute, 60596 Frankfurt, Germany; 7 Weizmann Institute, Rehovot, Israel; 8 Department of Medicine A, University of Münster, 48149 Münster, Germany; 9 Department of Surgery, Thoracic Surgery, Rems-Murr-Kliniken, 71364 Winnenden, Germany; 10 Department of Thoracic Surgery, Niels-Stensen-Kliniken, 49179 Ostercappeln, Germany; 11 Institute of Pathology, Martin-Luther-University Halle-Wittenberg, 06097 Halle (Saale), Germany; 12 National Center for Tumor Diseases (NCT), 69120 Heidelberg, Germany; 13 Present Address: Shanghai University of Medicine and Health Sciences, Shanghai, 201318 PR China; 14 Present Address: Department of Dermatology and Venereology, Martin-Luther-University Halle-Wittenberg, 06120 Halle (Saale), Germany; 15 Present Address: Klinik für Pneumologie, Beatmungsmedizin und Thorakale Onkologie, Klinikum Ingolstadt, 85049 Ingolstadt, Germany

**Keywords:** Non-small-cell lung cancer, Prognostic markers

## Abstract

Non-small cell lung cancer (NSCLC) is the leading cause of cancer death worldwide underlining the urgent need for new biomarkers and therapeutic targets for this disease. Long noncoding RNAs are critical players in NSCLC but the role of small RNA species is not well understood. In the present study, we investigated the role of H/ACA box small nucleolar RNAs (snoRNAs) and snoRNA-bound ribonucleoproteins (snoRNPs) in the tumorigenesis of NSCLC. H/ACA box snoRNPs including the NOP10 core protein were highly expressed in NSCLC. High levels of either *NOP10* mRNA or protein were associated with poor prognosis in NSCLC patients. Loss of NOP10 and subsequent reduction of H/ACA box snoRNAs and rRNA pseudouridylation inhibited lung cancer cell growth, colony formation, migration, and invasion. A focused CRISPR/Cas9 snoRNA knockout screen revealed that genomic deletion of SNORA65, SNORA7A, and SNORA7B reduced proliferation of lung cancer cells. In line, high levels of SNORA65, SNORA7A, and SNORA7B were observed in primary lung cancer specimens with associated changes in rRNA pseudouridylation. Knockdown of either SNORA65 or SNORA7A/B inhibited growth and colony formation of NSCLC cell lines. Our data indicate that specific H/ACA box snoRNAs and snoRNA-associated proteins such as NOP10 have an oncogenic role in NSCLC providing new potential biomarkers and therapeutic targets for the disease.

## Introduction

Lung cancer remains the leading cause of cancer death worldwide, with an estimated 1.6 million deaths each year [1]. Non-small cell lung cancer (NSCLC), the most common subtype with 85% of all cases, has an overall 5-year survival rate of 16%, which has not improved significantly for several decades [2]. Recent therapy approaches with targeted therapies and immunotherapy have somewhat improved the picture but the long-term outlook of advanced stage patients remains bleak. As lung cancer is a molecularly heterogeneous disease [3], an improved understanding of its biology and Achilles heels might lead to novel, effective therapies.

Noncoding RNAs (ncRNAs) have recently emerged as disease drivers in diverse human diseases including cancer [4]. Long ncRNAs (lncRNAs) such as MALAT1 are well recognized for important roles in lung cancer pathogenesis and prognosis prediction [5, 6]. Beyond lncRNAs, small RNA species exist with less well-understood functions in lung cancer. Small nucleolar RNAs (snoRNAs) constitute a group of intron-encoded ncRNAs, which range from 60 to 300 nucleotides in length. SnoRNAs are grouped into two families termed box C/D snoRNAs (SNORDs) and box H/ACA snoRNAs (SNORAs) [7, 8]. SnoRNAs are prominently located in the nucleolus and are required for posttranscriptional modifications of ribosomal RNA (rRNA). SnoRNAs often function as part of multicomponent protein complexes, collectively referred to as small nucleolar ribonucleoprotein (snoRNP) complexes [8]. The highly conserved box C/D snoRNAs, first described in the late 1980s [9], guide 2′-O-methylation at specific sites on the rRNA in complex with the methyltransferase fibrillarin, NOP56, NOP58, and the 15.5- kD/SNU13 protein. H/ACA box proteins together with H/ACA box snoRNAs guide up to 100 pseudouridine modifications on mammalian rRNAs. Each H/ACA snoRNP consists of a single guide H/ACA snoRNA and a protein complex comprised of the pseudouridine synthase dyskerin (DKC1) and the essential core components NOP10, NHP2, and GAR1. The association of DKC1, NOP10, and NHP2 with H/ACA snoRNAs appears to be essential for the biogenesis and formation of a catalytically active H/ACA snoRNP complex [10]. Recent findings in human cells suggest that pseudouridine residues within 28S rRNA may have a pivotal role in stabilizing rRNA thereby potentially impacting structure, protein composition, and function of the ribosome [11]. Recent studies suggest that H/ACA box snoRNAs are frequently altered in hematological disorders and solid tumors including lung cancer [10, 12–14]. Mutations in several genes encoding protein components of H/ACA box snoRNPs, such as DKC1 and NOP10, have been identified in cancer and congenital bone marrow failure syndromes [15–17]. Taken together, perturbation of H/ACA box snoRNA function and expression may contribute to cancer pathogenesis.

In this study, we systematically investigated the impact of H/ACA box snoRNAs/RNPs on tumorigenicity and their prognostic role in NSCLC. We identified, that increased expression of the H/ACA box protein NOP10 was associated with a poor prognosis of NSCLC patients and its deletion inhibited cell growth, proliferation, migration, and invasion of lung cancer cells via dysregulation of SNORA65, SNORA7A, and SNORA7B.

## Results

### The snoRNP complex protein NOP10 is highly expressed in NSCLC and is associated with a poor prognosis

**Fig. 1:**
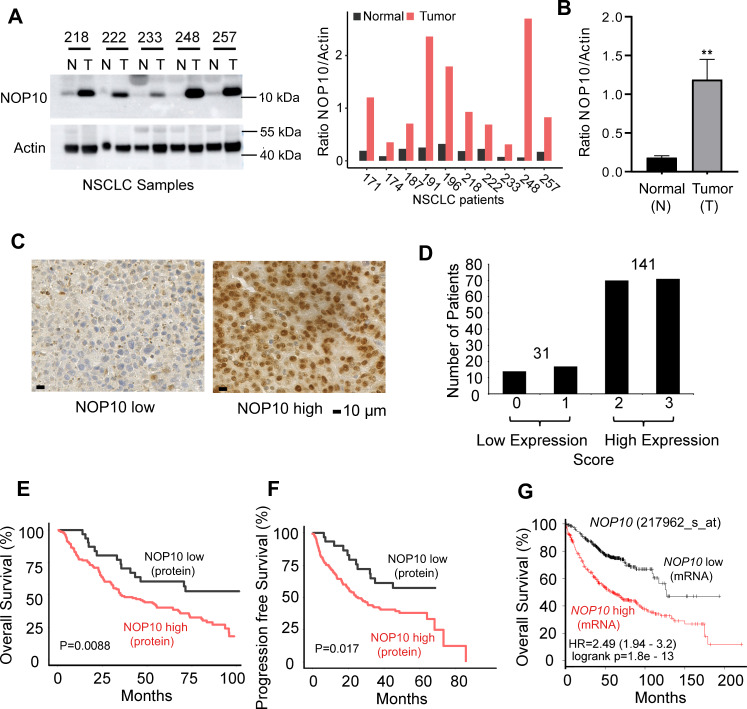
NOP10 is highly expressed in NSCLC patients and is associated with patients’ prognosis. **a** NOP10 protein levels in matched normal (N) and tumor (T) tissue as determined by western blot. Representative examples from five NSCLC patients are given. Western blots from additional five T/N pairs are provided in Supplementary Fig. 1a. Beta Actin levels are shown as loading control. All western blot images have been cropped for improved clarity and conciseness (left). Densitometrical analysis of western blot results from ten matched normal (N) and tumor (T) NSCLC samples (right). **b** Mean and SD of NOP10/Actin ratios of all normal (N) and tumor (T) samples depicted in **a** and Supplementary Fig. 1a (*n* = 10 per T and N samples, **p = 0.0012). **c** NOP10 immunohistochemistry staining of tumor samples from 172 NSCLC patients. Nuclear staining of NSCLC specimens was analyzed with QuPath software [50]. Representative positive and negative stainings are shown. Scale bars, 10 μm. **d** Bar graph providing numbers of patients with high and low NOP10 protein expression as analyzed by IHC. NOP10 protein expression is associated with poor overall (**e**, *p* = 0.0088) and progression-free (**f**, *p* = 0.017) survival of NSCLC patients. Kaplan–Meier plots are given for patients with low and high NOP10 protein expression. **g** *NOP10* mRNA expression is associated with poor overall survival in a published dataset of 720 adenocarcinoma patients (*p* = 1.8e−13, [51]). Kaplan–Meier plots are given for patients with low and high *NOP10* mRNA expression.

We evaluated the expression of H/ACA box snoRNP core proteins in NSCLC. NOP10 was overexpressed in ten primary NSCLC samples compared to paired normal lung tissue (*p* = 0.0012, Fig. 1a, b, Supplementary Fig. 1a). DKC1 and GAR1 were also expressed at high levels in lung cancer samples (Supplementary Fig. 1b). But, NHP2 was weakly expressed in lung cancer samples, when compared to paired normal lung tissue (Supplementary Fig. 1b). We further investigated the prognostic impact of NOP10 by immunohistochemistry in a large cohort of early stage NSCLC patients (*n* = 172, Supplementary Table 1). Substantial NOP10 protein expression was observed in 141 of the analyzed 172 patient samples (82%) (Fig. 1c, d) confirming the western blot results. High NOP10 protein levels were associated with poor overall survival (median 34.13 vs. 67.17 months, *p* = 0.0088, Fig. 1e) and worse progression-free survival (median 23.03 vs. 45 months, *p* = 0.017, Fig. 1f). This association was unrelated to patients’ sex, smoking status, age, tumor histology, or tumor grade (Supplementary Fig. 1c–h). We performed a multivariate Cox regression analysis, which included NOP10 expression status, age, tumor stage, tumor grade, smoking status, and sex. NOP10 remained an independent prognostic parameter in this multivariate analysis (HR NOP10 low = 0.33, *p* = 0.038, Supplementary Fig. 1i and Supplementary Table 2). We further analyzed the NSCLC TCGA dataset [18] with regard to NOP10 expression and driver mutations of NSCLC. Interestingly, EGFR mutations were associated with lower *NOP10* expression, whereas KEAP and RBM10 mutations were associated with higher *NOP10* levels (Supplementary Fig. 1j). These findings suggest that subtypes with different pathogenetic pathways may exhibit varying NOP10 levels.

We further substantiated the association of NOP10 expression and prognosis by using previously published gene expression data of 720 NSCLC patients with adenocarcinoma [19]. High levels of *NOP10* mRNA predicted worse overall survival (OS, median 59 vs. median 127 months, *p* < 0.001, Fig. 1g) as well as worse postprogression survival (PPS, median 14.1 vs. 25.8 months, *p* = 0.033, Supplementary Fig. 1k) and a shorter time to first progression (median 22.4 vs. 37.5 months, *p* = 0.018, Supplementary Fig. 1l). *GAR1* or *NHP2* mRNA expression did not correlate with patients’ survival (Supplementary Fig. 2a, b). For DKC1, the enzymatically active component of the H/ACA box snoRNP complex, there was a significant correlation between high *DKC1* mRNA levels and poor overall survival of NSCLC patients (median 59 (52) months vs. 77.6 (80.9) months, Supplementary Fig. 2c, d). Contrary to *NOP10, DKC1* mRNA expression was not associated with PPS (data not shown) indicating a less pronounced prognostic impact of *DKC1* expression in NSCLC.

Taken together, these data provide evidence that high levels of *NOP10* mRNA and protein predict a poor prognosis in NSCLC.

### NOP10 knockdown (KD) impairs proliferation, colony formation, migration, and invasion of NSCLC cells

**Fig. 2:**
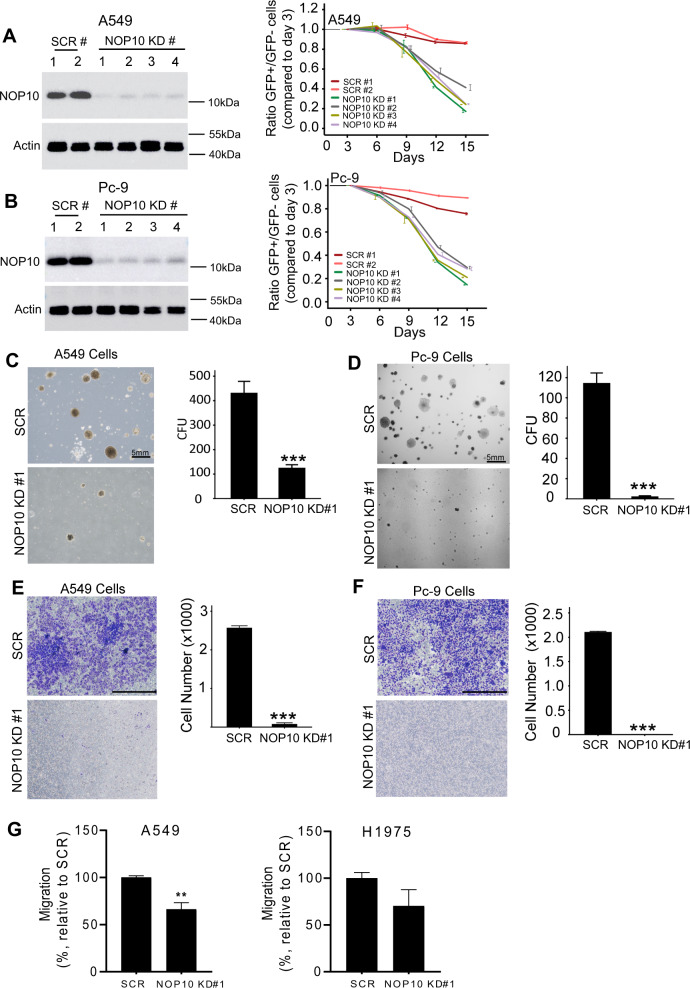
NOP10 knockout inhibits proliferation, colony formation, invasion, and migration capacity of NSCLC cells. **a**, **b** Western blot analysis of NOP10 in CRISPR/Cas9 NOP10 KD and scr control A549 (**a**, left) and Pc-9 cells (**b**, left). Actin served as loading control. Data are representative for three independent experiments. For proliferation analysis ratio of GFP-positive vs. GFP-negative A549 (**a**, right) and Pc-9 cells (**b**, right) was normalized to the ratio at day 3. Means ± S.D. are given for three independent experiments. **c** and **d** Representative microscopy images of colony formation assays using A549 scr control and NOP10 KD cells (**c**, left) and Pc-9 scr control and NOP10 KO cells (**d**, left). Number of Colony Forming Units (CFU) produced by scr control or NOP10 KO A549 (**c**, right) or Pc-9 (**d**, right) cells were counted. Data are represented as mean ± S.D. for three independent experiments (*p* < 0.001). **e, f** Transwell invasion assay for indicated NSCLC cell lines. NSCLC cells invading the Matrigel-coated membrane were stained by crystal violet. Representative images of A549 scr and NOP10 KO (**e**, left) and Pc-9 scr and NOP10 KO cells (**f**, left) are shown. Scale bar, 5 mm. Number of scr control and NOP10 KO cells invading the Matrigel membrane are given for A549 cells (**e**, right) and Pc-9 cells (**f**, right). Data are presented as mean ± S.D. for three independent experiments (*p* < 0.001). **g** Migration of A549 scr vs. NOP10 KD cells (left) and H1975 scr vs. NOP10 KD cells (right). Data are presented as mean ± S.D. for three independent experiments (**p < 0.01).

The prognostic impact of NOP10 mRNA and protein expression in NSCLC patients hinted at a functional role of NOP10 in NSCLC pathogenesis. Functional experiments using NSCLC cell lines were performed to test this hypothesis. We generated NOP10 KDs using CRISPR/Cas9 in the NSCLC cell lines A549, Pc-9, H1975, and H358, all displaying endogenously high levels of NOP10 protein (Supplementary Fig. 3a). Sanger sequencing showed that transduction with a CRISPR/Cas9 expression construct and small gRNAs (sgRNAs) targeting NOP10 induced genomic mutation in NSCLC bulk cells (Supplementary Fig. 3b). Western blot analysis demonstrated depletion of NOP10 in the NSCLC cell lines upon transduction with the CRIPSR/Cas9 KD construct (Fig. 2a, b, and Supplementary Fig. 3c, d). Loss of NOP10 inhibited proliferation of all NSCLC cell lines compared to scramble (scr) control cells (Fig. 2a, b and Supplementary Fig. 3c, d) implicating a general role of NOP10 in NSCLC proliferation, independent of the respective cell line. Thus, all further experiments were mainly performed with A549 and Pc-9 cells, widely used in biomedical studies and representing model cell lines for KRAS-mutation (A549) and EGFR-mutation (Pc-9), the two most frequently identified oncogenic drivers in NSCLC [20].

NOP10-depleted A549 and Pc-9 cells were impaired in clonogenic growth with reduced colony numbers and colony size compared to scr control A549 and Pc-9 cells (*p* < 0.001, Fig. 2c, d and Supplementary Fig. 3e). We further investigated the impact of NOP10 on migration and invasion capacity of NSCLC cells. Transwell cell culture inserts were coated with Matrigel and invasion assays were performed using A549 and Pc-9 cell lines with and without NOP10 KD. The invasion capacity of A549 and Pc-9 cells with NOP10 KD was significantly suppressed compared to that of scr control cells (*p* < 0.001, Fig. 2e, f and Supplementary Fig. 3g). Similar results were obtained when migration capacity was analyzed. NOP10 KD significantly reduced migration of A549 and Pc-9 cells compared to the respective scr controls (*p* < 0.01, Fig. 2g). Thus, our results indicate that NOP10 is required for proliferation, migration, and invasion of lung cancer cells.

### H/ACA box snoRNAs are highly expressed in NSCLC patient samples and can be depleted by KD of NOP10

**Fig. 3:**
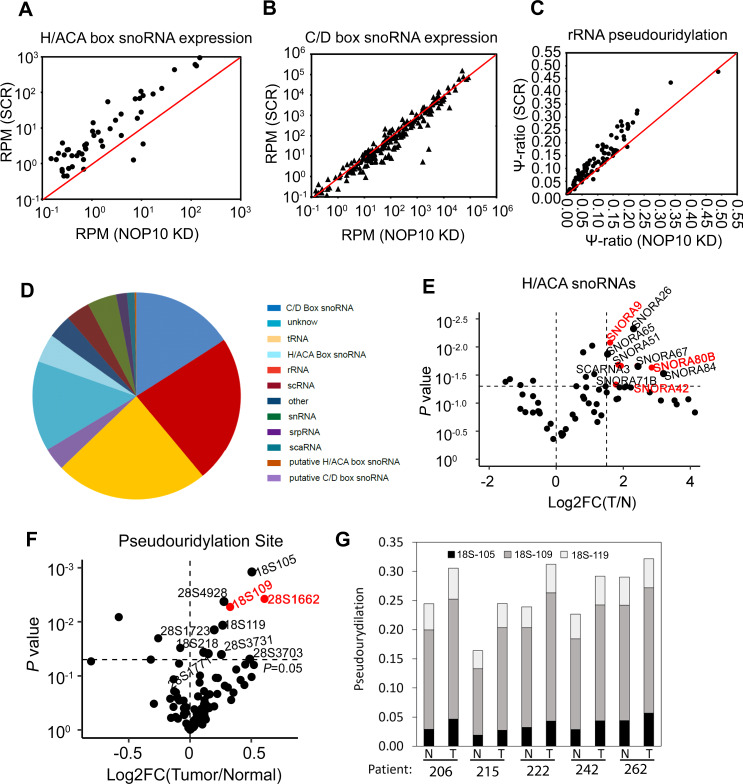
NOP10 depletion reduces H/ACA box snoRNAs which are highly expressed in primary lung cancer samples. **a, b** SnoRNA-Seq was performed in scr control and NOP10 KO A549 cells. Scatterplots present H/ACA box snoRNA expression (**a**) and C/D box snoRNA expression ratios (**b**) (*p* < 0.01, paired Wilcoxon test). **c** Changes in rRNA pseudouridylation upon NOP10 depletion. Scatter plot presents pseudouridylation ratios of 18S and 28S rRNAs in scr control and NOP10 KO A549 cells (*p* < 0.01, paired Wilcoxon test). **d** Distribution of snoRNA sequence reads and different RNA species with percentages of the total mapped reads. Data are presented as pie chart. **e** Volcano plot indicates the log fold change in H/ACA box snoRNA expression (*x*-axis) and *p* value (*y*-axis) in patients’ tumor (T) vs. normal (N) tissue (*n* = 7). The horizontal dashed line denotes a *p* value of 0.05. The vertical dashed line represents log2 FC (T/N) > 1.5. Upregulated snoRNAs marked in red promote higher pseudouridylation of their target sites in T vs. N (as depicted in **f**). **f** Volcano plot indicates the log fold change in pseudouridylation levels (*y*-axis) and *p* value (*x*-axis) in patients’ tumor (T) vs. normal (N) tissue (*n* = 5). The horizontal dashed line denotes a *p* value of 0.05. Pseudouridylation sites marked in red are targets of snoRNAs enriched in T vs. N (see **e**, depicted in red). Target rRNA positions for individual snoRNAs have been annotated based on MODOMICS [49, 52]. SnOPY [53] annotations are given in Supplementary Table 4 (colum 3). **g** Pseudouridylation levels at a hotspot region in 18S rRNA with differences between lung cancer and matched normal lung tissue (*p* = 0.026, paired *t*-test for sum of pseudouridylation levels). The stacked bar diagram shows the pseudouridylation levels at three closely related sites in 18S rRNA (individual paired *t*-test *p* values: 18S-105: *p* = 0.004; 18S-109: *p* = 0.105; 18S-119: *p* = 0.062).

NOP10 is an essential component for H/ACA box snoRNP formation and function. The H/ACA box snoRNP complex can stabilize snoRNAs and thus regulate snoRNA expression levels. We established and performed medium-sized (40–200 nucleotides) RNA sequencing to quantify snoRNAs (hereafter termed snoRNA-Seq [14]) in NSCLC cells. Depletion of the H/ACA box snoRNP component NOP10 decreased H/ACA box snoRNA abundance in A549 cells, but, as expected, C/D box snoRNAs were not depleted (*p* < 0.001, Fig. 3a, b).

SnoRNAs act as guides for posttranscriptional modifications of rRNA with H/ACA box snoRNAs guiding pseudouridylation. In human rRNA, 95 uridines are predicted to be modified to pseudouridine in 28S, 18S, 5.8S, and 5S rRNAs [21]. To evaluate the impact of NOP10 on this specific rRNA modification, we mapped pseudouridylation throughout the entire rRNA sequence in scr control and NOP10 KD A549 cells using pseudouridylation-sequencing (Ψ-seq) [22, 23]. Loss of NOP10 severely reduced global rRNA pseudouridylation (*p* < 0.01, Fig. 3c). Beside its role in 18S rRNA production and rRNA pseudouridylation, the four H/ACA snoRNP proteins are also components of the telomerase complex, bind the lncRNA *TERC* via its conserved H/ACA domain and are required for telomere maintenance [24]. Thus, we analyzed whether NOP10 depletion also affected expression levels of *TERC*. Loss of NOP10 modestly reduced *TERC* expression as compared to scr control (Supplementary Fig. 4a). However, by analyzing TCGA NSCLC patients’ data [18, 25], we did not detect a correlation between *NOP10* expression and the expression of telomerase components (Supplementary Fig. 4b, c).

To analyze the expression of snoRNAs in NSCLC patients, we performed snoRNA-Seq with RNA from seven matched tumor and normal lung specimens. SnoRNA-Seq captured 309 snoRNAs. Human lung specimens expressed a higher number of C/D box snoRNAs compared to H/ACA box snoRNAs (16% mapped reads for 242 box C/D snoRNAs and 4.4% mapped reads for 67 box H/ACA snoRNAs) (Fig. 3d). Several H/ACA box snoRNAs were significantly enriched in lung cancer samples compared to paired normal lung tissue (*p* < 0.05, Fig. 3e and Supplementary Table 3). We also performed Ψ-seq for the entire rRNA sequence in five matched tumor and normal NSCLC specimens to identify changes in pseudouridylation of rRNAs. We identified statistically increased pseudouridylation levels in ten H/ACA box snoRNA target sites of the rRNA (*p* < 0.05, Fig. 3f and Supplementary Table 4) in all tumor samples compared to matched controls. Changes in rRNA pseudouridylation levels occurred throughout the 18S and 28S rRNA with one region each in 18S and 28S rRNA where several adjunct uridines were affected. In 18S rRNA, pseudouridylation at positions 105, 109, and 119 of 18S rRNA were all increased in tumor specimens compared to matched normal lung tissue (Fig. 3g). Similar findings were obtained for the region encompassing nucleotides 3700–3732 of 28S rRNA which also showed increased pseudouridylation of four uridines (Supplementary Fig. 3f).

### H/ACA box snoRNAs are required for growth of NSCLC cells

**Fig. 4:**
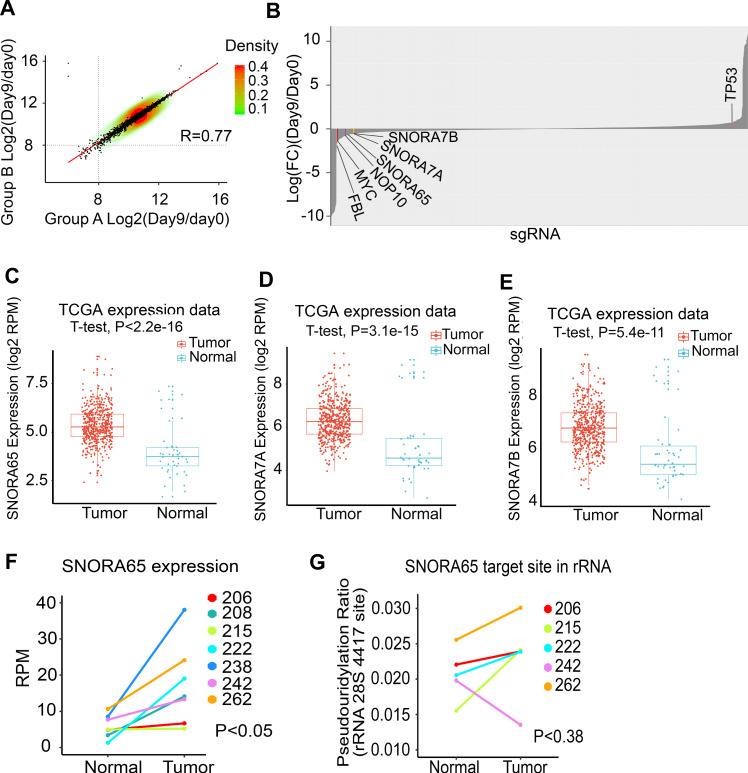
H/ACA box snoRNAs are crucial for NSCLC growth. **a** Replicate comparison of gRNA levels in CRISPR/Cas9 snoRNA KO library-transduced A549 cells (day 9/day 0). The log2 values for all gRNAs targeting snoRNAs and choosen genes in the library are plotted and fit to a linear model. *R* indicates Pearson’s correlation. A strong correlation between the two independent experiments (group A and group B) is demonstrated (*R* = 0.77). **b** Positively and negatively selected snoRNAs in A549 cells after 9 days posttransduction with a pooled snoRNA CRISPR/Cas9 KO library. Data are presented as waterfall plot. **c**–**e** Expression levels of SNORA65 (**c**), SNORA7A (**d**), and SNORA7B (**e**) in the TCGA NSCLC patient cohort comparing tumor and matched normal tissue. **f** SNORA65 expression was significantly increased in tumor tissue compared to matched normal tissue as identified by snoRNA-Seq (*p* < 0.05). **g** Pseudouridylation of 28S rRNA 4417 targeted by SNORA65. Pseudouridylation levels are increased in tumor tissue compared to matched normal tissue in four out of five analyzed patients (*p* < 0.38).

To reveal the role of individual snoRNAs on cell growth in lung cancer, a CRISPR/Cas9 snoRNA knockout screen with a pooled LentiCRISPR library was performed in A549 cells. We designed a snoRNA library targeting 283 known human snoRNAs and additional control genes as well as genes coding for snoRNP proteins (Supplementary Fig. 5 and Supplementary Table 5). For each snoRNA and protein-coding gene up to six guide RNAs (gRNAs) were included in the library, which totally consisted of 1559 gRNAs. For most snoRNAs only 2–3 gRNAs were found. Transduced A549 cells were selected after 3, 5, and 9 days posttransduction to identify snoRNAs that either positively or negatively affected cell growth and viability. PCR-amplified barcode gRNA regions from the extracted genomic DNA of cells before and after CRISPR screening were subjected to deep-sequencing analysis (Supplementary Fig. 5). We performed two independent experimental replicates (group A and group B) which showed close correlation (Fig. 4a, *R* = 0.77). The screen enriched for positive control gRNAs targeting TP53, a well-described tumor suppressor [26] (Fig. 4b). Depletion of gRNAs targeting the negative control MYC, a prominent oncogene in a diversity of cancers [27] (Fig. 4b) indicated that proliferation- relevant genes could be identified by the screen. We also identified depletion of gRNAs targeting NOP10, again demonstrating that NOP10 is necessary for growth and survival of NSCLC cells (Fig. 4b). We used the model-based analysis of genome-wide CRISPR/Cas9 knockout algorithm [28] to identify the top hits by comparing samples from days 5 and 9, respectively, with day 0 controls (serving as a representation of all gRNAs in the library). In total, we identified 20 negatively selected and 26 positively selected gRNAs targeting specific snoRNAs with statistical significance (FDR ≤ 0.8; Supplementary Table 6). Our analyses indicated that SNORA65, SNORA7A and SNORA7B (as reflected by the depletion of the respective targeting gRNAs, Fig. 4b and Supplementary Table 6) could promote growth and proliferation of lung cancer cells. We further analyzed the expression of SNORA65, SNORA7A, and SNORA7B using the TCGA NSCLC data sets [18, 25].

TCGA data demonstrated that SNORA7A (*p* = 3.1e−15), SNORA7B (*p* = 5.4e−11), and SNORA65 (*p* < 2.2e−16) are highly expressed in tumor samples compared to normal non-tumor matched controls (Fig. 4c–e). In line, significantly increased levels of SNORA65 (*p* < 0.05) were also found in tumor samples of our own snoRNA-Seq experiments (Fig. 4f). In addition, we identified overexpression of SNORA7A in four out of seven tumors compared with the matched normal tissues (Supplementary Fig. 6a). Five out of seven tumor samples showed SNORA7B overexpression (Supplementary Fig. 6b). For SNORA7A and 7B, the differences were not statistically significant for all seven matched samples, probably due to limited sample size. Of note, increased rRNA pseudouridylation levels were found for 28S 1771, the target site of SNORA7A /7B (Fig. 3f, Supplementary Fig. 6c and Supplementary Table 4). Also, four of the five tumor samples showed higher 28S 4417 pseudouridylation levels, the target site of SNORA65, compared to matched normal tissues when analyzed in detail (Fig. 4g). Overall, these findings indicate that snoRNA expression correlates with pseudouridylation of the respective rRNA target sites in NSCLC tumor samples. Interestingly, target sites of SNORA65, SNORA7A, and SNORA7B are all located around the ribosomal peptidyl transferase center (Supplementary Fig. 6d), which is characterized by the most pronounced accumulation of universally conserved rRNA nucleotides in the entire ribosome [29].

### SNORA65, 7A, and 7B are required for growth and proliferation of lung cancer cells

**Fig. 5:**
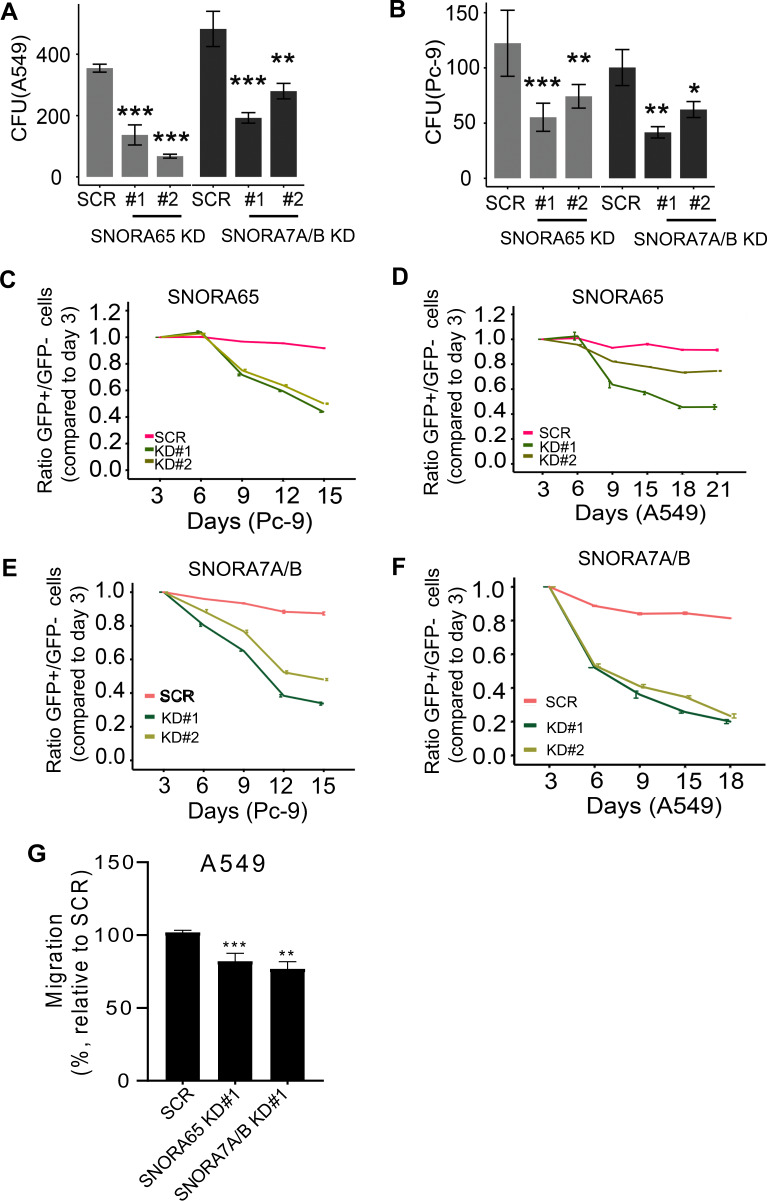
Depletion of SNORA65, SNORA7A, and SNORA7B decreases colony formation capacity and proliferation of NSCLC cells. **a, b** Number of colonies formed by A549 (**a**) or Pc-9 (**b**) scr control and SNORA65, SNORA7A, or SNORA7B KD cells. Data are presented as mean ± S.D. from three independent experiments. **c**–**f** Pc-9 cells (**c**, **e**) and A549 cells (**d**, **f**) were infected with lentiviruses expressing scr control gRNA and gRNAs targeting SNORA65 or snoRA7A/B. Percentage of infected GFP-positive cells was analyzed using flow cytometry. Ratio of GFP-positive vs. GFP-negative cells at days 6, 9, 12, 15, 18, and 21 post infection was normalized to the ratio at day 3. Data are presented as mean ± S.D. from three independent experiments. **g** Migration of A549 scr vs. SNORA65A and SNORA7A/B KD cells, respectively, (**p < 0.01, ***p = 0.0007). Data are presented as mean ± S.D. from three independent experiments.

We further analyzed single H/ACA box snoRNA functions in NSCLC. We generated KDs of SNORA65 and SNORA7A/7B in A549 and Pc-9 cells using CRISPR/Cas9 for the respective introns (Supplementary Fig. 7a). Sanger sequencing of buk cells showed that gRNAs targeting SNORA65 and SNORA7A/B induced genomic mutation, and significant loss of snoRNA expression was detected by q-RT-PCR (Supplementary Fig. 7b–e). Importantly, CRISPR/Cas9*-*mediated snoRNA KD did not impair the expression of the host gene RPL32 (Supplementary Fig. 7f). Depletion of SNORA65 and SNORA7A/7B significantly impaired clonogenic growth of A549 cells and Pc-9 cells (*p* < 0.05, Fig. 5a, b). Further, depletion of SNORA65 and SNORA7A/7B significantly inhibited proliferation of A549 and Pc-9 cells as determined by evaluating the growth rate of cancer cells at different time points after transduction (*p* < 0.05, Fig. 5c–f). As for NOP10, loss of SNORA65 and SNORA7A/B reduced migration of A549 cells (Fig. 5g). To confirm specificity and the functional impact of SNORA65A and 7A/B onto proliferation and growth of NSCLC cells, we depleted an unrelated H/ACA box snoRNA, which was not significantly altered in NSCLC as determined by our CRISPR/Cas9 library screen. KD of SNORA31 did not impair proliferation nor colony formation capability of NSCLC cells (Supplementary Fig. 8a–e) demonstrating that specific snoRNAs, but not a nonspecific random dysregulation of snoRNAs, affect growth of NSCLC cells.

### Downregulation of SNORA65, 7A, and 7B neither affect cell cycle progression nor apoptosis of NSCLC cells

Suppression of SNORA65 and SNORA7A/7B inhibited growth and proliferation of lung cancer cells. For single snoRNAs such as SNORD76, it could be demonstrated that growth and proliferation of glioma cells were affected by arresting the cancer cells in S-phase of the cell cycle thus inhibiting tumorigenicity [30].

**Fig. 6:**
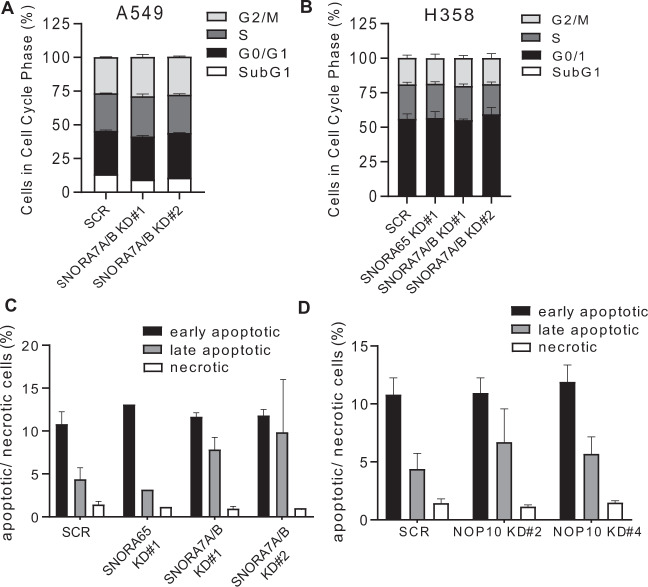
Manipulation of H/ACA box snoRNAs does not affect cell cycle nor induces apoptosis of NSCLC cells. **a**–**b** Cell cycle phase distribution in **a**, A549 scr control and SNORA7A7B KD cells and in **b**, H358 scr, SNORA65, and SNORA7A/B KD cells as analyzed by PI staining and flow cytometry. Data are presented as mean ± S.D. from three independent experiments. **c–d** A549 scr, SNORA65 KD, and SNORA7A/B KD cells (**c**) and A549 scr and NOP10 KD cells (**d**) were stained with annexin V/7-AAD and percentage of apoptotic cells was analyzed by flow cytometry. Data are presented as mean ± S.D. from three independent experiments.

Thus, we analyzed the influence of SNORA65 and 7A/7B depletion on cell cycle phase distribution in nocodazole-synchronized A549 and H358 cells. PI staining with flow cytometry analysis showed that depletion of SNORA65 and SNORA7A/7B did not affect percentage of cells in G0/G1, S, and G2/M phases as compared to scr controls (Fig. 6a, b). Number of cells in SubG1, a marker for apoptosis, was also not changed (Fig. 6a, b). In line, the percentage of apoptotic and necrotic cells did not differ in SNORA7A/B KD and scr control cells when analyzed by annexin V/7-AAD staining (Fig. 6c). Further, KD of NOP10 also did not induce apoptosis of NSCLC cells (Fig. 6d).

Thus, our results indicate that depletion of SNORA65 and SNORA7A/7B inhibits proliferation and tumorigenicity of lung cancer cells which does not associate with immediate apoptosis or cell cycle arrest.

## Discussion

During the past 20 years, important progress has been made in our understanding of the molecular pathogenesis of lung cancer, whose malignant transformation has been demonstrated to result from the accumulation of genetic aberrations such as in EGFR [31], KRAS [32], and ALK [33]. However, little light has been shed on nonprotein coding RNAs, except for microRNAs, which are well characterized by target recognition and regulatory functions in cancer [34, 35]. While the study of microRNAs in lung cancer received a lot of attention over the last decade, highly efficient therapeutic options or the establishment of diagnostic methods based on noncoding RNAs are still lacking. Recent data suggest an important role of medium-size and long-noncoding (lnc) RNAs in the pathogenesis and prognosis of lung cancer [36–38]. In 2011, we could already describe tumor-promoting functions of the lncRNA MALAT1 in NSCLC and a direct association with patient’s survival [6]. The first report that highlighted the pathological importance of the medium-sized snoRNAs demonstrated that H5sn2 (a box H/ACA snoRNA) was distinctly downregulated in meningioma [39]. Further, SNORD50 was reported to have a tumor suppressive role in breast and prostate cancer [40, 41], while SNORA42 was reported to act as an oncogene in lung and colorectal cancer [36, 42]. Beside the snoRNAs itself, the proteins of the snoRNP complex could be dysregulated or mutated in cancer. Here, dyskerin (DKC1) is the best investigated and most prominent example. Dyskerin mutations and subsequently decreased rRNA pseudouridylation are characteristic of X-linked dyskeratosis congenita, an inherited bone marrow failure syndrome [43], and patients display a clear increase in susceptibility to cancers [44]. For the other components of the H/ACA box snoRNP, only little data exist so far regarding their dysregulation in cancer. Thus, our study aimed to investigate both, the role of the snoRNP protein components as well as the role of snoRNAs itself in the pathogenesis of NSCLC to define new biomarkers and therapeutic targets urgently needed to improve patients’ outcome. We identified a significant upregulation of NOP10 protein in NSCLC tumor tissue compared to matched normal controls in the adenocarcinoma as well as in the squamous subtype. In vitro, NOP10 suppression could inhibit cell growth, proliferation, and tumorigenicity of lung cancer cell lines. We also identified an overexpression of DKC1, the catalytically active component of the H/ACA box snoRNP in NSCLC tumor samples. However, the association of DKC1 expression with survival (OS, PFS) of NSCLC patients was not that pronounced as for NOP10, neither in the adenocarcinoma nor in the squamous cell subtype. As it is reported that all four components of the H/ACA box snoRNP complex are required for stability and function of the complex [45], a deregulation of one component such as NOP10 would be sufficient for the deregulation of the whole complex, affecting the stability of snoRNAs. In line, our snoRNA-Seq experiments demonstrated that H/ACA box snoRNAs and subsequent rRNA pseudouridylation were significantly downregulated in NSCLC cells upon NOP10 KD. Using snoRNA-Seq of seven primary NSCLC patient samples, we identified an overexpression of individual H/ACA box snoRNAs in matched tumor vs. normal tissue. Of the 309 well-characterized captured snoRNAs in our snoRNA-Seq approach, nine H/ACA box snoRNAs were significantly enriched in the NSCLC tumor samples compared to matched normal controls. Among these we also identified SNORA42 to be upregulated in NSCLC patients. This is well in line with other published data showing that SNORA42 is commonly increased in a number of solid tumors including lung cancer and that high SNORA42 expression in NSCLC patients correlated with poor survival [36]. We additionally identified SNORA65, 7A, and B to be upregulated in NSCLC patients in our snoRNA-Seq experiments, a finding not yet reported. The upregulation of SNORA65, 7A, and B in tumor tissue was confirmed using the NSCLC TCGA dataset. Importantly, using a snoRNA knockout library screen, we also identified SNORA65, SNORA7A, and SNORA7B as regulators of cell growth in NSCLC. Individual suppression of these snoRNAs inhibited proliferation of NSCLC cells. Zhang et al. recently reported that SNORA7A functioned by inducing snoRNP formation by binding DKC1 and subsequently catalyzing pseudouridines in 28S rRNA [46]. Thus, it is most likely that snoRNAs act through snoRNPs to regulate tumor behavior. However, additional snoRNA functions beside the guidance of pseudouridylation cannot be ruled out yet.

Further, the core proteins of H/ACA snoRNPs are also structural components of the human telomerase complex [24] and increased telomerase activity has been reported as a marker for poor prognosis in NSCLC [47]. However, we only observed a modest reduction in *TERC* levels, the RNA component of the telomerase complex, upon NOP10 KD and there was no association between *NOP10* expression and expression of telomerase genes using the NSCLC TCGA dataset. However, we cannot exclude that NOP10 also contributes to NSCLC pathogenesis by affecting telomerase function.

In summary, our study identified NOP10 and the associated H/ACA box snoRNAs SNORA65, 7A, and 7B as important growth regulators in NSCLC cells, representing potential new biomarkers for the disease. Given that cancer cells often show perturbation at the translational level, snoRNAs and snoRNPs are likely to contribute to tumorigenesis by affecting ribosomes and protein translation. An exciting avenue for future studies lies in identifying the detailed downstream functional consequences of deregulated SNORA65, SNORA7A, and SNORA7B expression and in developing therapeutic strategies to target H/ACA box snoRNAs in NSCLC.

## Materials and methods

### Cell lines

293T cells were cultured in Dulbecco’s modified Eagle’s medium (Thermo Fisher Scientific, Schwerte, Germany) supplemented with 10% fetal bovine serum (Biochrom, Berlin, Germany) and 1% penicillin/streptomycin (P/S) (Sigma-Aldrich, Taufkirchen, Germany). Human A549, Pc-9, H358, and H1975 cells were cultured in RPMI 1640 medium (Thermo Fisher Scientific, Schwerte, Germany) supplemented with 10% FBS and 1% P/S. All cells were maintained in a humidified incubator at 37 °C and 5% CO_2_.

Cell lines were purchased from Deutsche Sammlung von Mikroorganismen und Zellkulturen GmbH (DSMZ, Braunschweig, Germany) and have been tested to be free of mycoplasma contamination.

### Cloning

The pL-CRISPR.EFS.GFP vector expressing SpCas9 and sgRNAs for the relevant target snoRNAs and target genes, respectively, has been described previously (Addgene, plasmid no. 57818). For cloning of gRNAs directed against human NOP10 and individual snoRNAs, oligos were annealed and inserted into the Esp3I digested vector. All vectors were confirmed by sequencing before use. gRNA sequences are provided in Supplementary Table 7.

### Sanger sequencing

To confirm the CRISPR/Cas9-mediated target gene KD, genomic regions around the gene-specific CRISPR/Cas9 target site were amplified by PCR and analyzed by Sanger sequencing. Primer sequences are provided in Supplementary Table 8.

### CRISPR-Cas9 gRNA library screen

A CRISPR-Cas9 snoRNA library with 1559 sgRNAs targeting known snoRNAs, snoRNA host genes, as well as positive (MYC, RUNX1, EIF4A1) and nontargeting controls was designed (see Supplementary Table 5 and Supplementary Fig. 5). The library sgRNAs were cloned into the lentiCRISPRv2 vector (Addgene, plasmid no. 52961). Screening was performed with slight modifications to the protocol of Shalem et al. [48].

### snoRNA-Seq

SnoRNA-Seq was conducted as described previously [14] using matched tumor and normal samples from seven NSCLC patients.

### rRNA Ψ-seq

Ψ-seq was performed on total RNA as described [22] using matched tumor and normal samples from five NSCLC patients already analyzed in snoRNA-Seq. Reads were aligned to the rRNA using Bowtie and processed as described [22]. For each site annotated as containing a Ψ in MODOMICS [49, 53], a Ψ-ratio was calculated, capturing the number of reads beginning at the position (that is, reflecting termination of reverse transcriptase) divided by the number of reads overlapping it.

### Statistical analysis

For statistical analyses, SPSS version 22 (IBM, Ehningen, Germany), R studio 3.5.0, and GraphPad Prism 6 (GraphPad Software, San Diego, CA) were used. Data were analyzed for normal distribution before statistical analyses and for variance between groups. Values are presented as mean ± S.D. or mean ± SEM of replicates. Student’s *t* test or one-way ANOVA analysis were used to determine statistical significance unless stated otherwise. For Kaplan–Meier survival analysis, the log-rank test was used to determine statistical significance. *p* values < 0.05 were considered to be statistically relevant (*p < 0.05; **p < 0.01; ***p < 0.005).

## Supplementary information

Supplementary Material

Supplementary Tables

## Data Availability

snoRNA-seq and Ψ-seq data are stored at NCBI’s Gene Expression Omnibus data repository with the accession code GSE161232. Further materials and methods are available from “Supplementary Material”.
