# Supplementary material for: NOP10 predicts lung cancer prognosis and its associated small nucleolar RNAs drive proliferation and migration
Source: Oncogene. 2020 Dec 7;40(5):909–21. doi: 10.1038/s41388-020-01570-y (PMC7862062; doi:10.1038/s41388-020-01570-y)
Supplement: Supplementary file 1 — Supplementary Material [file 41388_2020_1570_MOESM1_ESM.pdf]

Supplementary Figures, Legends and Supplementary Materials and Methods

NOP10 predicts lung cancer prognosis and its associated small nucleolar RNAs drive proliferation and migration

Chunhong Cui, Yi Liu, Dennis Gerloff, Christian Rohde, Cornelius Pauli, Marcel Köhn, Danny Misiak, Thomas Oellerich, Schraga Schwartz, Lars-Henning Schmidt, Rainer Wiewrodth, Alessandro Marra, Ludger Hillejan, Frank Bartel, Claudia Wickenhauser, Stefan Hüttelmaier, Stefanie Göllner, Fengbiao Zhou, Bayram Edemir and Carsten Müller-Tidow

Suppl. Figure 1, associated with Figure 1 (NOP10 is highly expressed in NSCLC patients and is associated with patients' prognosis.) (A) NOP10 protein levels in matched normal (N) and tumor (T)- tissue as determined by western blot. Representative examples from five NSCLC patients are given. Actin levels are shown as loading control. All western blots images have been cropped for improved clarity and conciseness. Western blots from additional five T/N pairs are provided in Figure 1A. **(B)** Immunoblotting of protein extracts from matched normal (N) and tumor (T)-tissue was performed to detect DKC1, NHP2 and GAR1 protein levels. Actin levels are shown as loading control. Five representative N/T sample pairs are shown. Due to limitations in primary sample material, DKC1, NHP2, GAR1 (Suppl. Fig. 1B) and NOP10 (Fig. 1A) were all detected on the same membrane using membrane stripping and reprobing. Thus, Actin levels provided are the same for Suppl. Fig. 1B and Fig. 1A. **(C-H)** Association of NOP10 protein expression with sex **(C)**, smoking behavior **(D)**, age **(E)**, malignancy **(F)**, histology **(G)** and tumor grade **(H)**. **(I)** Multivariate analysis of HRs for Overall Survival (OS) with $p < 0.05$ is shown. HRs are presented as the

means (95 % CI). **(J)** Correlation of *NOP10* expression with tumor mutational burden in NSCLC (TCGA data set). **(K)** Post Progression Survival (PPS) and **(L)** First Progression of adenocarcinoma patients (n=125) [1]. Kaplan Meier Plots are shown for patients with high and low *NOP10* mRNA expression (http://kmplot.com/analysis/).

Supplementary Figure 1

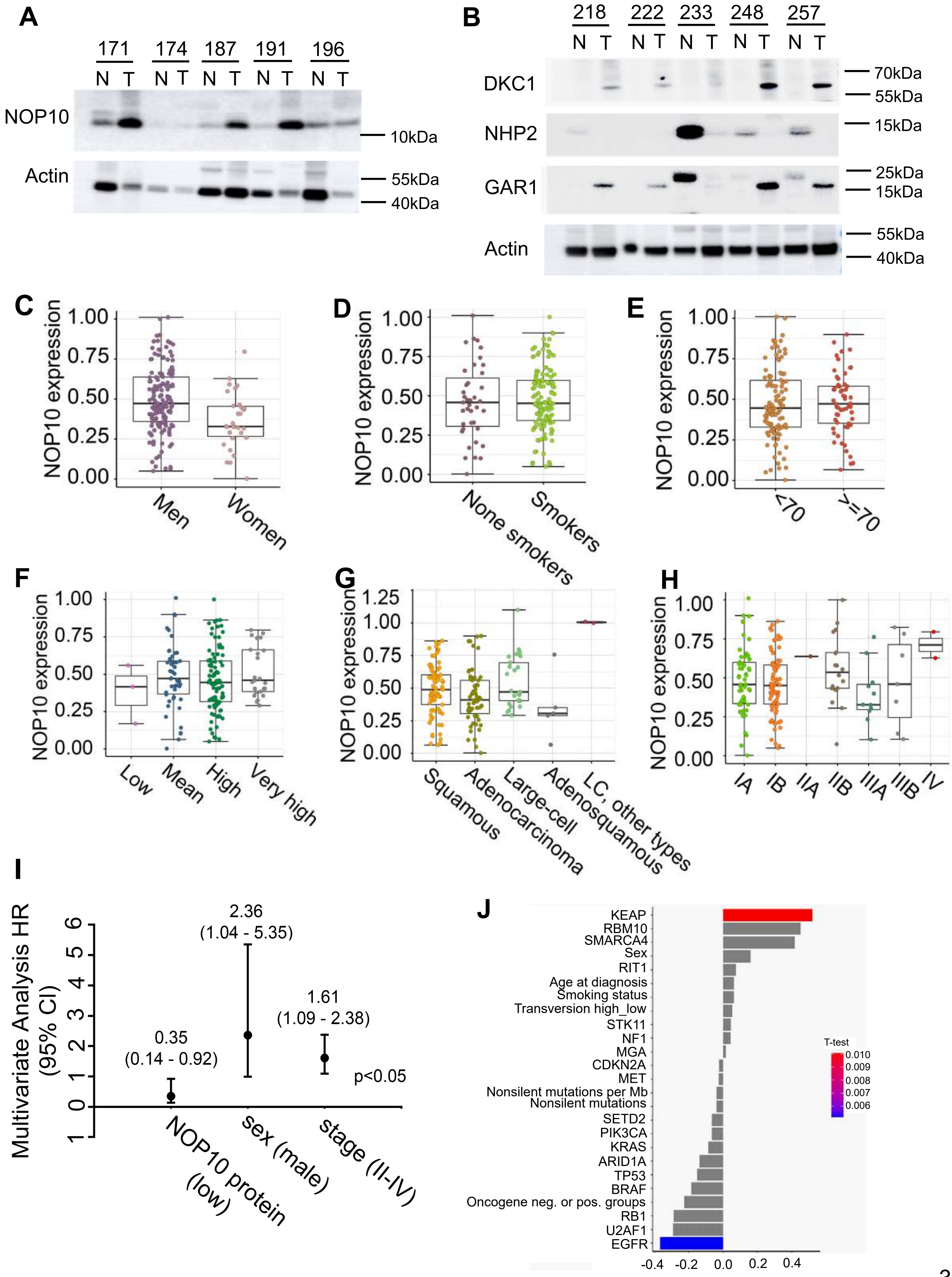

Supplementary Figure 1, continued

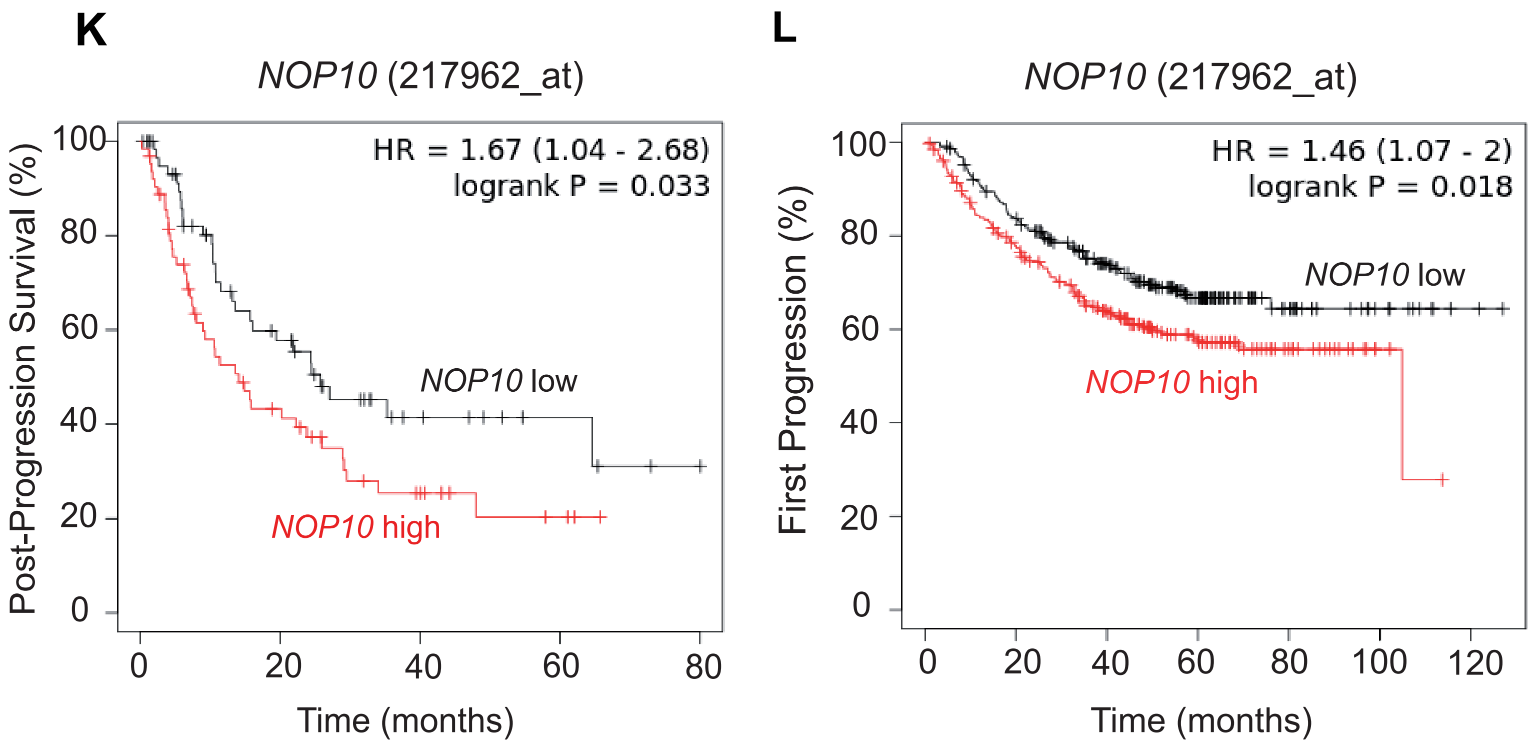

Suppl. Figure 2, associated with Figure 1 (NOP10 is highly expressed in NSCLC patients and is associated with patients' prognosis. (A and B) Overall survival for adenocarcinoma patients (n=720) [1] with low and high *GAR1* **(A)** and *NHP2* **(B)** mRNA expression. Kaplan–Meier plots are given (http://kmplot.com/analysis/). **(C and D)** Overall Survival for adenocarcinoma and squamous carcinoma patients (n=1925) with low and high DKC1 mRNA expression. Data from two different available Affymetrix probes are shown. Kaplan–Meier plots are given (http://kmplot.com/analysis/).

Supplementary Figure 2

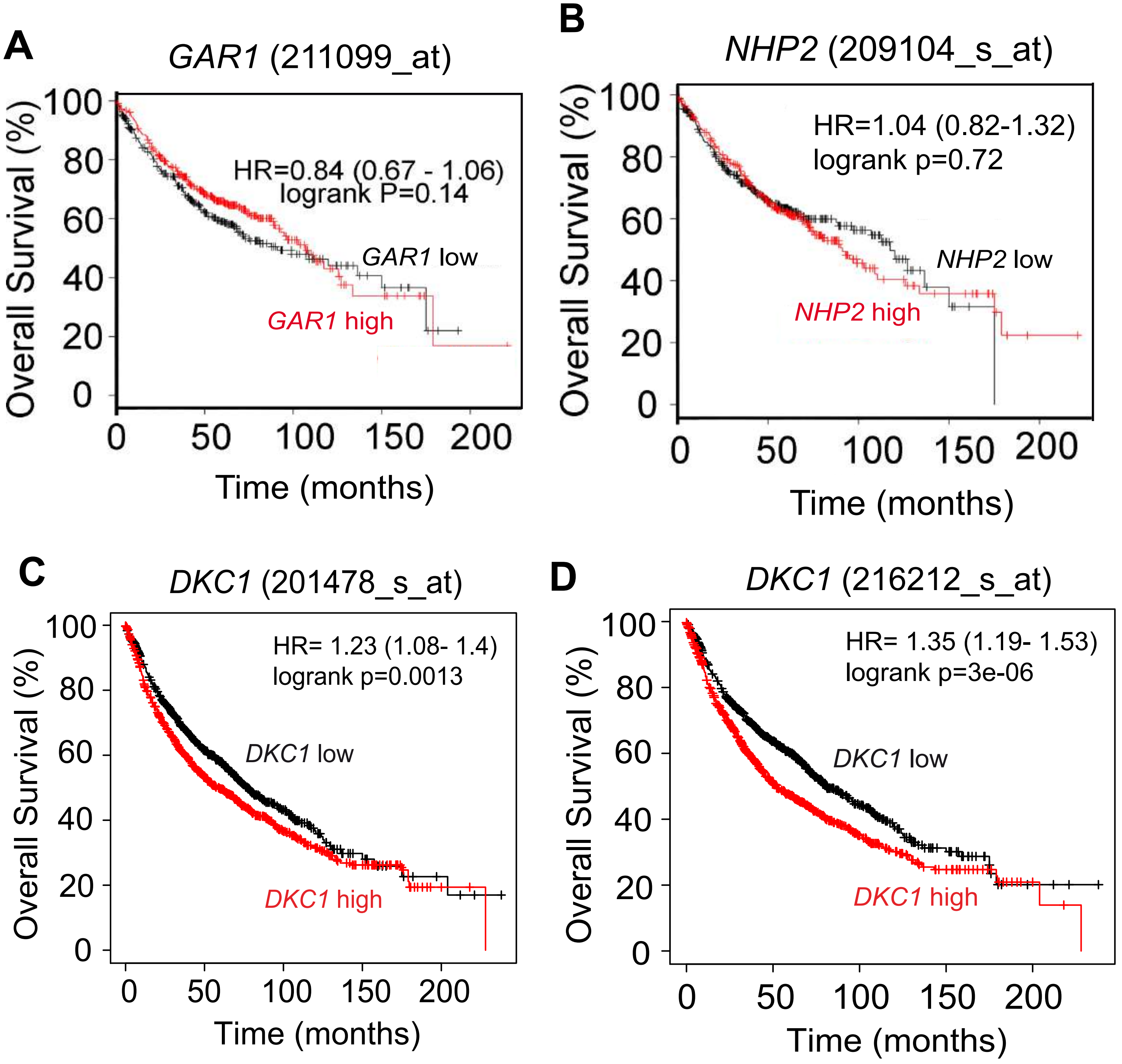

Suppl. Figure 3, associated with Figure 2 (NOP10 knockdown inhibits proliferation, colony formation, invasion and migration capacity of NSCLC cells.) **(A)** NOP10 protein expression in all NSCLC cell lines used within the study compared to normal non-malignant control (HSAEC cells). Actin is used as loading control. Results are representative for two independent experiments. **(B)** Sequencing chromatogram displaying genomic mutations introduced into the NOP10 genomic sequence by CRISPR/Cas9 sgRNA#1 in A549 (top) and Pc-9 (bottom) cells. **(C) and (D)** H1975 and H358 cells were infected with lentiviruses expressing scr control gRNA or different gRNAs targeting NOP10. Western blot analysis in bulk H1975 cells **(C, left)** and H358 cells **(D**, **left)** demonstrates efficient depletion of NOP10. Actin is used as loading control. Data are representative for three independent experiments. Ratio of infected, GFP-positive cells vs. uninfected GFP-negative cells was analyzed at the indicated time points (days post-infection). Ratio of GFP-positive vs. GFP-negative H1975 (**C, right**) and H358 cells (**D, right**) at days 6, 9, 12, 15 and 18 post-infection was normalized to the ratio at day 3. Means ±S.D. are given for three independent experiments. **(E)** CFU assays using scr control and NOP10 CRISPR/Cas9 KO Pc-9 cells. Representative microscopy images for two scr controls and four NOP10 gRNAs are given (top). Scale bar= 5mm. Numbers of colony-forming units for scr control and NOP10 KD cells. Mean and SD from three independent experiments are given (bottom). **(F)** Pseudouridylation levels at a hotspot region in 28S rRNA with differences between lung cancer and matched normal lung tissue (p= 0.025, paired t-test for sum of pseudouridylation levels). The stacked bar diagram shows the pseudouridylation levels at four closely related sites in 28S rRNA (individual paired t-test p-values: 28S-3732: p= 0.005; 28S-3728: p= 0.014; 28S-3704: p= 0.164, 28S-3700: p= 0.04). **(G)** Transwell invasion assay for indicated scr and NOP10 KD NSCLC cell lines. NSCLC cells invading the matrigel-coated membrane were stained by crystal violet.

Representative images of A549- and Pc-9- scr and *NOP10* KD cells are shown. Scale bar, 5 mm (top). Number of scr control and *NOP10* KD cells invading the matrigel membrane are given for A549 cells and Pc-9 cells (bottom). Data are presented as mean ±S.D. for three independent experiments ($p < 0.001$).

Supplementary Figure 3

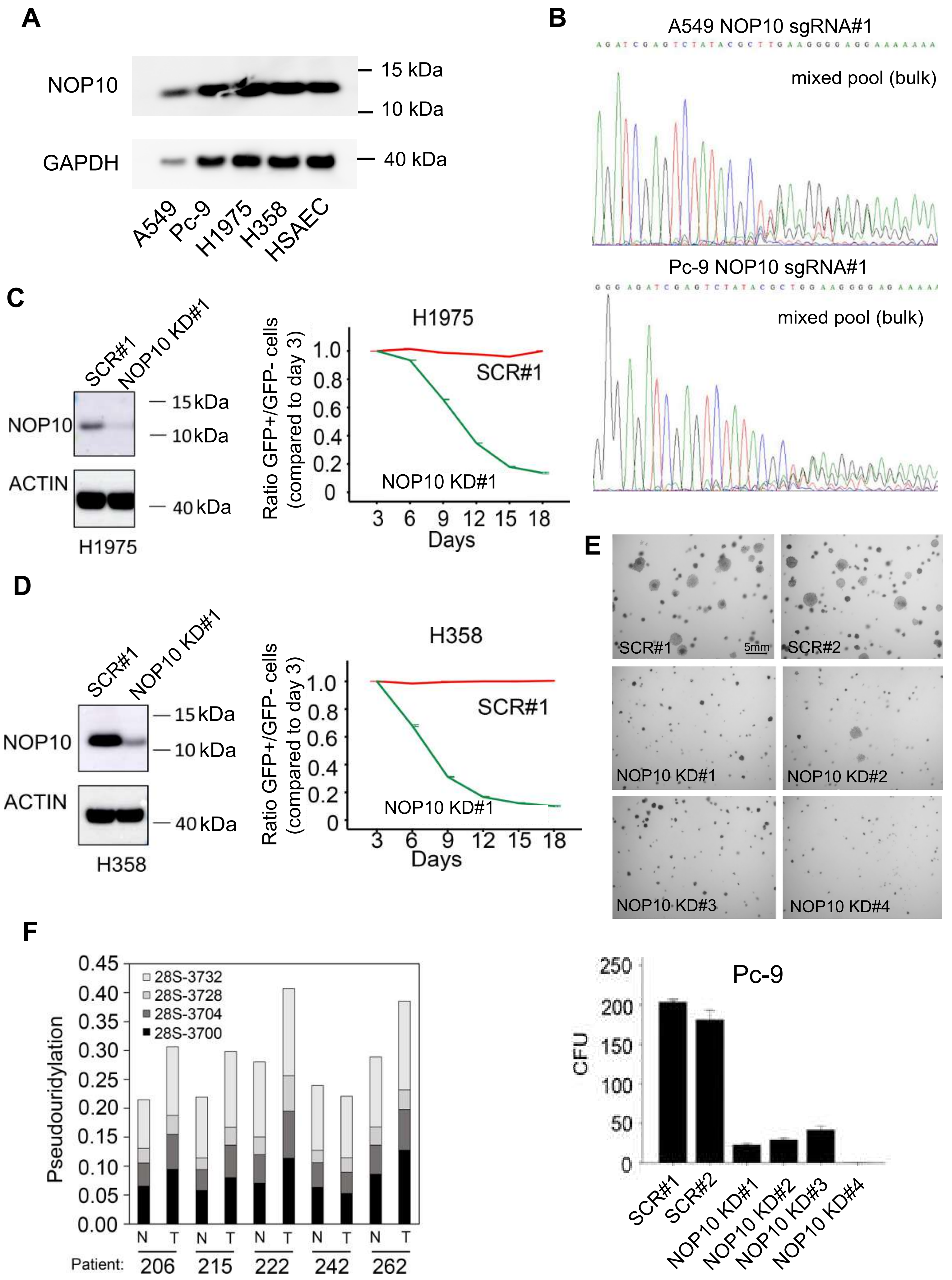

Supplementary Figure 3, continued

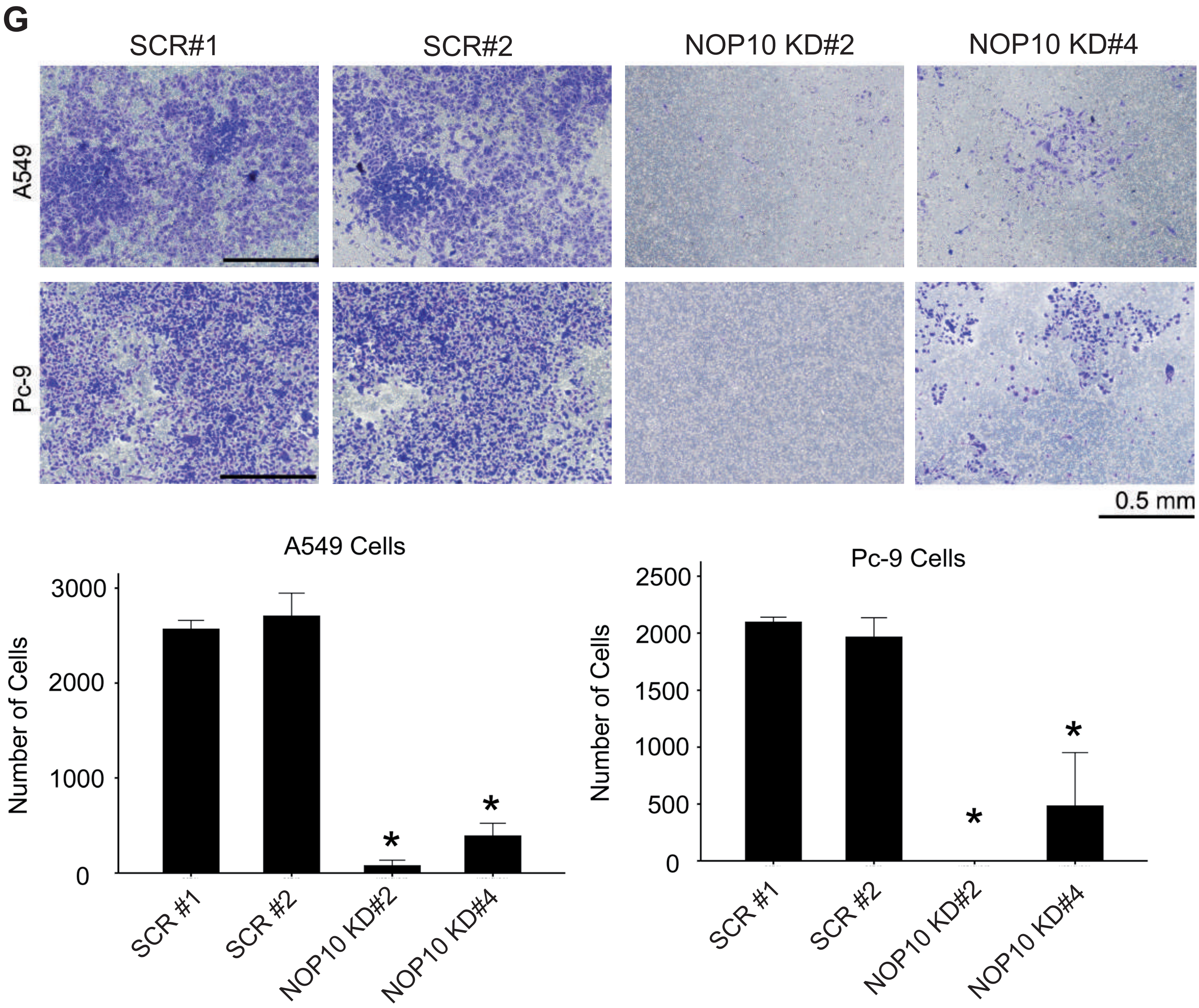

Suppl. Figure 4, associated with Figure 2 (*NOP10* knockdown inhibits proliferation, colony formation, invasion and migration capacity of NSCLC cells). **(A)** TERC mRNA expression in A549- and H358- NOP10 knockdown cells compared to scr control as estimated by RT-qPCR. GAPDH was used for normalization. Data are presented as mean ±S.D. from two independent experiments with 2-4 technical replicates (****p< 0.0001). **(B and C)** Correlation analysis for *NOP10* and TERT *mRNA* expression **(B)** as well as *NOP10* and *WRAP53* mRNA expression **(C)** using the TCGA NSCLC data set [2, 3].

Supplementary Figure 4

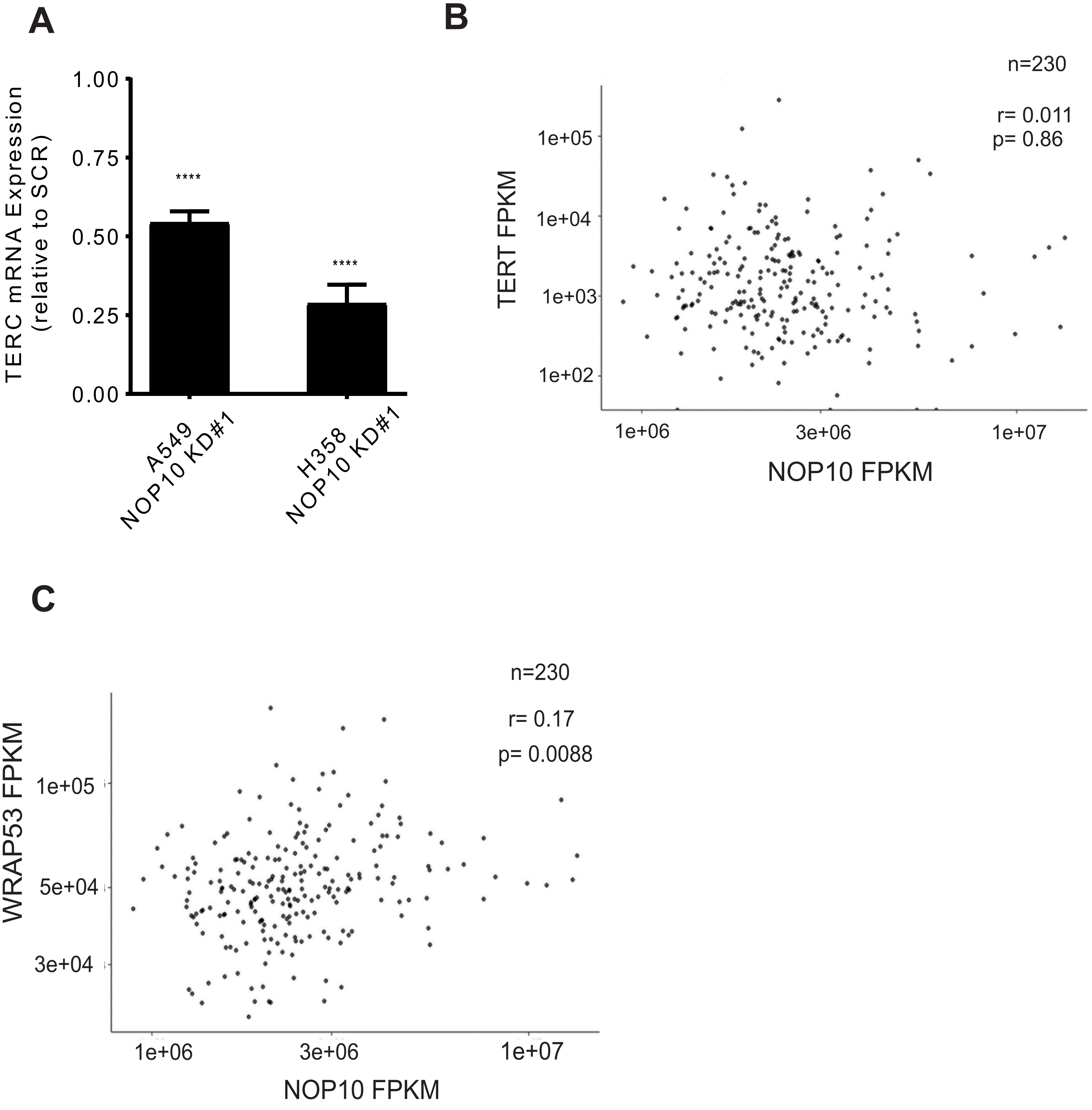

Suppl. Figure 5, associated with Figures 3 and 4 (H/ACA box snoRNAs are crucial for NSCLC growth):

Schematic overview of the CRISPR/Cas9 snoRNA knockout library approach

Supplementary Figure 5

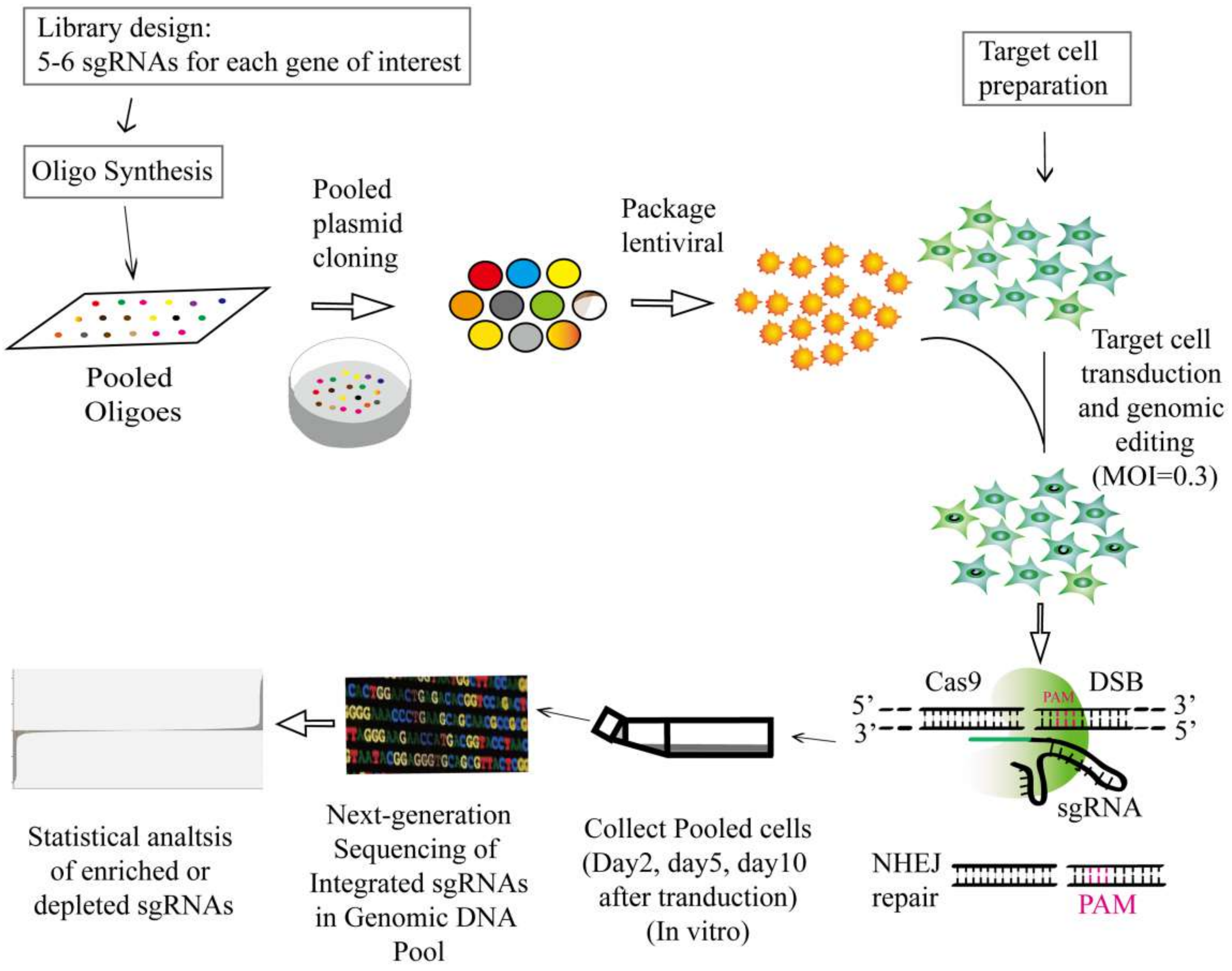

Suppl. Figure 6, associated with Figure 4 (H/ACA box snoRNAs are crucial for NSCLC growth). (A) SNORA7A expression levels are significantly increased in four out of seven tumor samples compared to matched normal tissue as identified by snoRNA-Seq ($p < 0.28$). **(B)** SNORA7B expression levels are significantly increased in five out of seven tumor samples compared to matched normal tissue as identified by snoRNA-Seq ($p < 0.72$). **(C)** Pseudouridylation of 28S rRNA site 1771 targeted by SNORA7A/B. Pseudouridylation levels are increased in tumour tissue compared to matched normal tissue in four out of five analysed patients ($p < 0.05$). **(D)** Depiction of the ribosomal peptidyl transferase centre including target sites of SNORA65, SNORA7A and SNORA7B

Supplementary Figure 6

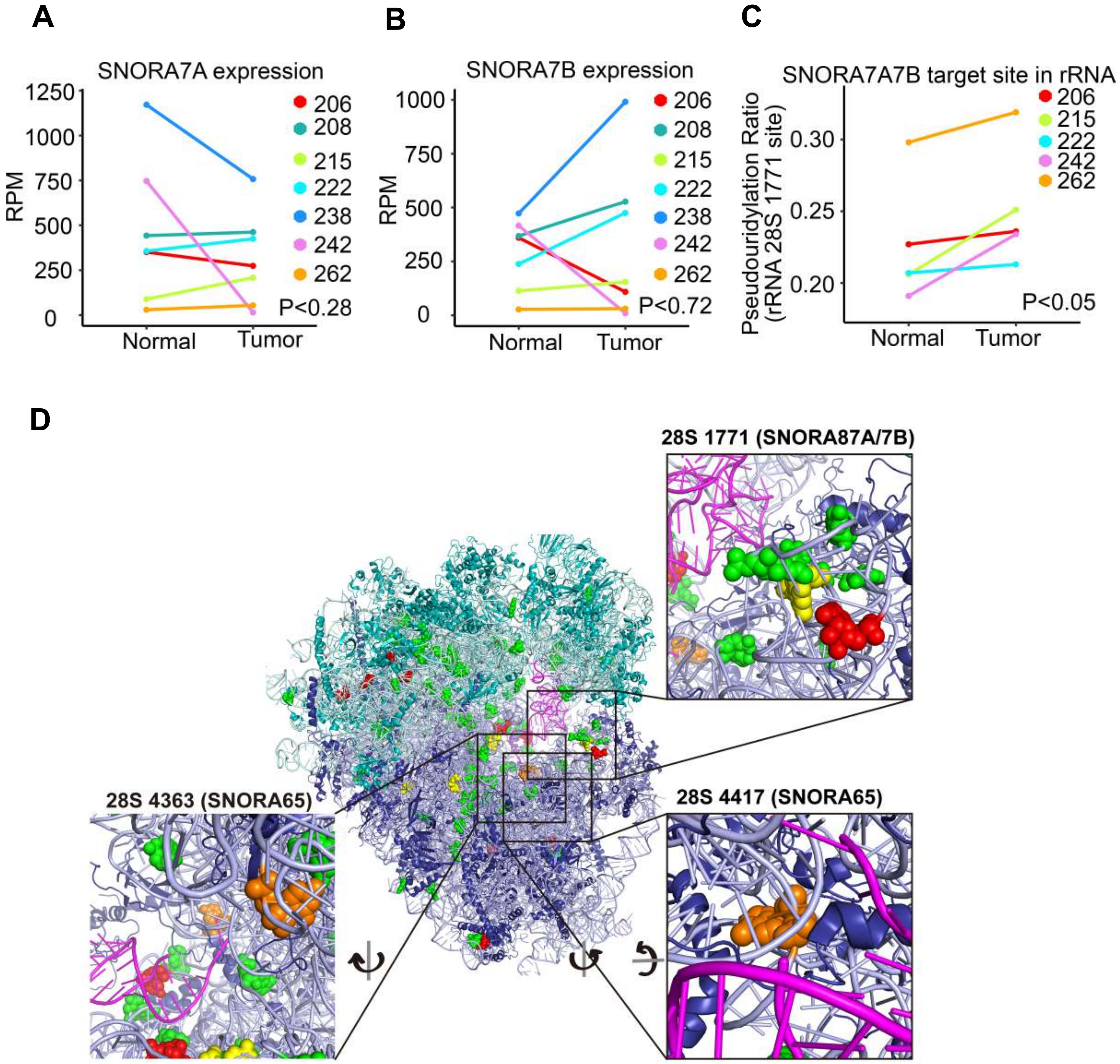

Suppl. Figure 7, associated with Figure 5 (Depletion of SNORA65, SNORA7A and 7B decreases proliferation and colony formation of NSCLC cells.) (A) Schematic overview of the CRISPR/Cas9 strategy to knockout SNORA65, SNORA7A and SNORA7B. **(B and D)** Sequencing chromatogram displaying genomic mutations introduced into the SNORA65 (**B**) and SNORA7A/B (**D**) genomic sequence by CRISPR/Cas9. **(C and E)** Expression of SNORA65 **(C)** and SNORA7A/B **(E)** was analyzed in Pc-9 scr control and KO cells by quantitative RT-PCR. **(F)** RPL32 protein levels in A549 (left) and Pc-9 (right) scr control and SNORA7A/7B KD cells as estimated by western blotting. Actin is used as loading control. Data are representative for two independent experiments.

Supplementary Figure 7

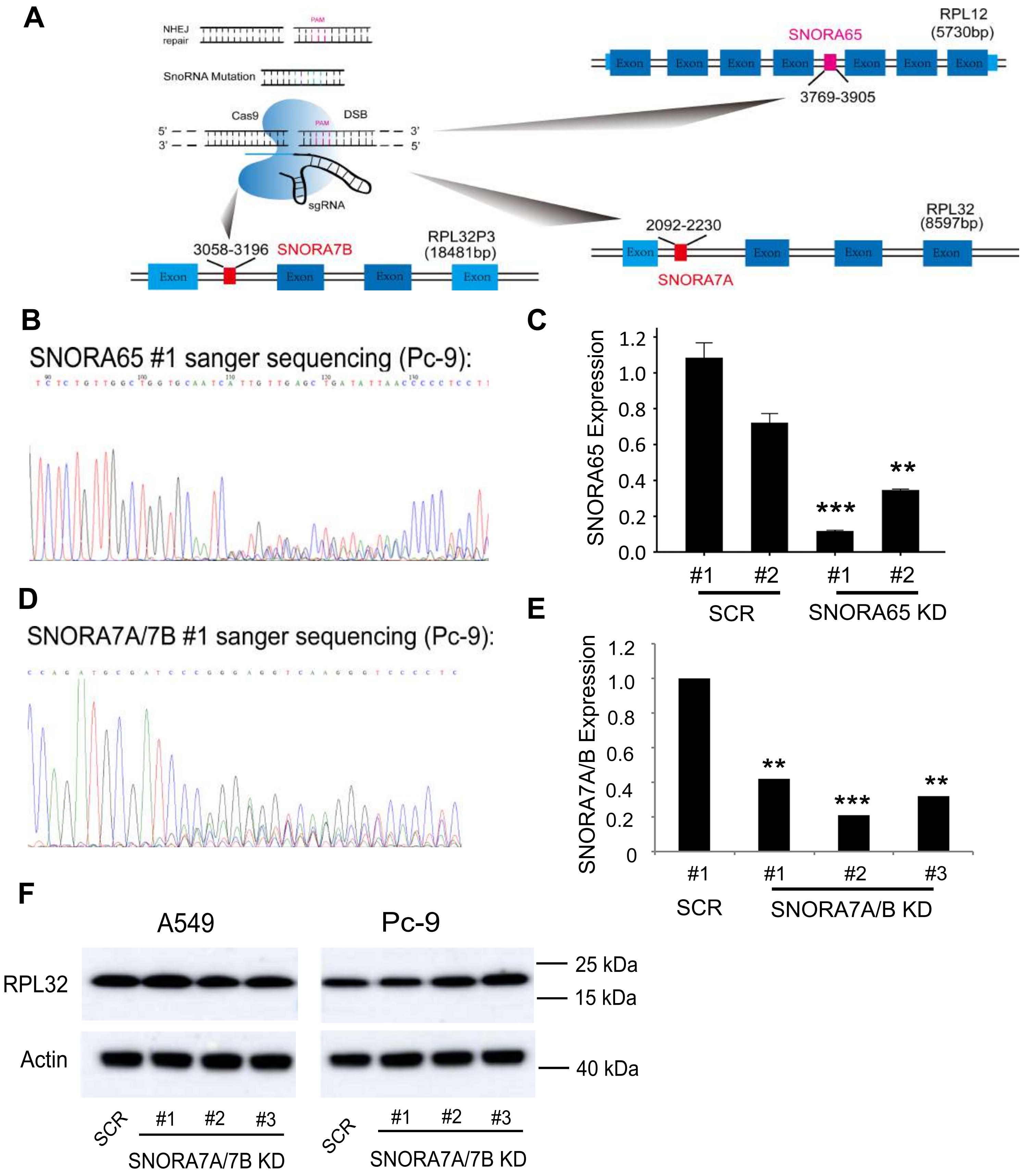

Suppl. Figure 8, associated with Figure 5 (Depletion of SNORA65, SNORA7A and 7B decreases proliferation and colony formation of NSCLC cells.) (A and B) RT-qPCR analysis of SNORA31 expression in A549 **(A)** and Pc-9 **(B)** scr control and SNORA31 KD cells. Data are presented as mean ±S.D. from two independent experiments with three technical replicates each. **(C and D)** Percentage of GFP-positive vs. GFP-negative scr and SNORA31 KD A549 (**C**) and Pc-9 cells (**D**) at days 6, 9, 12, 15 and 18 post-infection was normalized to the ratio at day 3. Means ±S.D. are given for three independent experiments. **(E)** Number of Colony Forming Units (CFU) produced by scr control or SNORA31 KD A549 cells. Data are presented as mean ±S.D. for three independent experiments.

Supplementary Figure 8

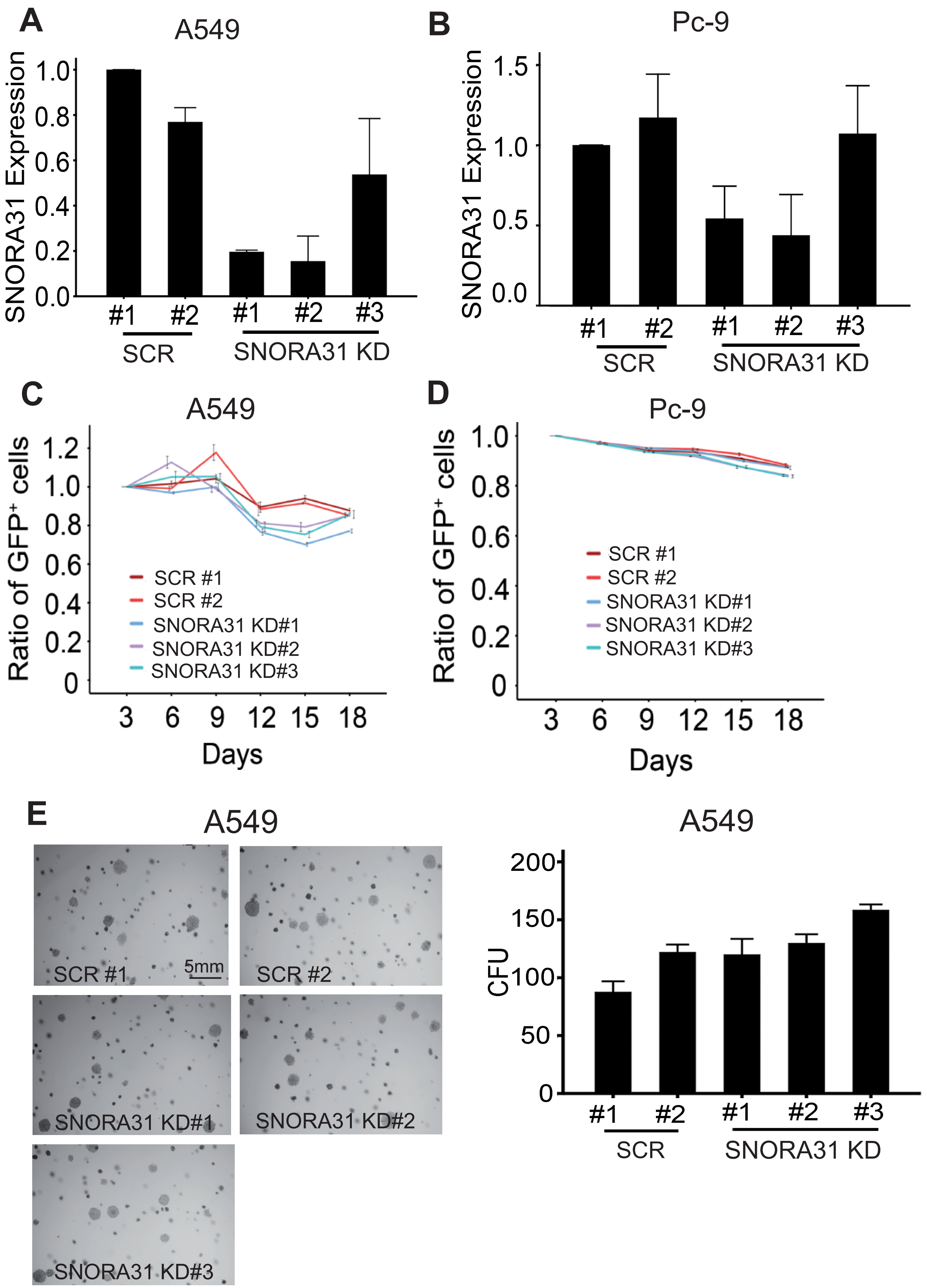

Supplementary Materials and Methods

Clinical samples and immunohistochemistry

Clinical samples and performance of immunohistochemistry stainings have been previously described [4]. Only patients with complete follow-up data were included in the statistical analysis. According to the WHO classification of lung tumors, the primary pulmonary lesion was classified as 81 squamous cell carcinomas (47%), 59 adenocarcinomas (34.3%), 25 large cell carcinomas (14.5%) and 7 unspecified cases (4.1%).

Written informed consent was obtained from all patients and the study was approved by the local Institutional Review Board. The study was in compliance with all applicable national and local ethics guidelines. The staining intensity of cells was scored 0 (no staining), 1 (weak staining), 2 (moderate staining) or 3 (strong staining) according to Remmele's Immunoreactive Score (IRS) [5]. To discriminate between low and high NOP10 expression, scores 0 and 1 were considered as low, scores 2 and 3 were considered as high expression. Baseline characteristics of patients are given in Supplementary Table 1.

Lentiviral transfection and transduction

293T cells were transfected with 10 µg target CRISPR/Cas9 plasmid and ViraPower helper plasmids (Life Technologies, Darmstadt, Germany) using TurboFect Reagent (Thermo Fisher Scientific, Schwerte, Germany). Supernatants containing viral particles were harvested 72h after transfection and virus particles were concentrated by ultracentrifugation. Lentiviral transduction of human NSCLC cell lines was performed in the presence of 8 µg/ml polybrene (Sigma-Aldrich, Taufkirchen, Germany). For all subsequent experiments, transduced bulk cells were used. For experiments involving

pL-CRISPR.EFS.GFP (single snoRNA knockdowns and NOP10 knockdown) transduced bulk cells were monitored for GFP expression by flow cytometry and either directly used in subsequent assays (CCG assay) or GFP-positive cells were sorted and subsequently analyzed (qRT-PCR, western blot, CFU assay, migration assay, invasion assay, apoptosis assay, cell cycle assay).

Competitive cell growth (CCG) assay

CCG assays to monitor the effect of gene knockdown onto cell proliferation were performed as previously described [6]. Briefly, NSCLC cell lines were infected with lentiviruses carrying scramble (scr) control gRNA, NOP10-targeting or snoRNA-targeting gRNA. Unsorted bulk cells (transduced GFP^+ and untransduced GFP^- cells) were used for the assay. Three days after infection, the percentage of transduced cells (GFP^+) was measured every three days for 5 times using flow cytometry. Proliferation rates were calculated by comparing percentage of GFP+ cells at the respective time points compared to day 3.

Colony formation assay

A total of 3.3×10^3 transduced, GFP positive human NSCLC cells were added to 3 ml RPMI1640 medium supplemented with 10% FBS, 1% P/S and 0.5% soft agar (Lonza, Rockland, ME USA). Cells were seeded in 6-well plates and cultured at 37°C, high humidity, and 5% CO_2. Colonies were counted after two weeks of growth without staining.

Migration Assay

Migration assays were performed as described [7].

Transwell invasion assay

A total of $1x10^4$ cells (in 100µl medium) were seeded into the upper part of a 24 well transwell chamber with matrigel-coated filter inserts with 6.5 mm diameter and a pore size of 8 µm (Corning Incorporated, Corning, NY). In the lower part of the chamber 750µl RPMI1640 supplemented with 10% FBS was added and the assay was incubated in a humidified tissue culture incubator at 37°C and 5% CO_2 atmosphere. The bottom medium was changed every day. After three days, cells on the lower level of the membrane passing the Matrigel matrix were stained with crystal violet (Sigma-Aldrich, Taufkirchen, Germany). All assays were performed in triplicate with three independent experiments.

Genomic DNA isolation

For isolation of genomic DNA from cultured cells, DNase Blood and Tissue kit from QIAGEN (Hilden, Germany) was used according to manufacturer's instruction.

Quantitative RT- PCR

For quantitative RT-PCR, RNA was isolated with Trizol according to standard protocols. A total of 0.5 µg RNA from each sample was reverse-transcribed using random hexamer primer and Moloney murine leukemia virus reverse transcriptase (M-MLV, ThermoFisher Scientific, Berlin, Germany), according to the manufacturer's guidelines. SYBR-Green PCR master mix (Applied Biosystems Deutschland GmbH, Darmstadt, Germany) containing 333 nM of each forward(F) and reverse(R) primer

(Supplementary Table 8) and 2 µl reverse-transcribed template were transferred into a 96-well PCR plate (ABgene, Hamburg, Germany). PCR was performed using a CFX96 Touch™ Real-Time PCR Detection System (BioRad, München, Germany). Melting curve analysis was used to monitor specificity of the PCR products. PCR for each sample was performed in triplicate. Negative controls without template were always included. GAPDH or Actin were used as internal control for the normalization of mRNAs. U6 or 5.8s rRNA was used to normalize snoRNAs. Relative quantification of gene expression was performed using the comparative threshold method [8]. Primer Sequences are given in Supplementary Table 8.

mRNA expression data sets

Published mRNA expression data sets of lung cancer patients [1] deposited online were analyzed using Kaplan-Meier -Plotter (KMP) (http://kmplot.com/analysis/).

Western blot analysis

Cells were lysed in RIPA lysis buffer (50 mM Tris-HCL [pH 8], 150 mM NaCl, 1% NP-40, 0.5% sodium deoxycholate, 0.1% SDS, protease inhibitors) for 30 min at 4 °C. Protein extracts were resolved by SDS–PAGE (4-12%, ThermoFisher Scientific, Berlin, Germany), blotted to nitrocellulose membranes (GE Healthcare, München, Germany) and probed with the following antibodies: Rabbit anti-NOP10 (Abcam, ab133726), rabbit anti-DKC1 (Abcam, ab64667), rabbit anti-NHP2 (Abcam, ab172481), rabbit anti-Gar1 (Abcam, ab188617), rabbit anti-RPL32 (Abcam, ab229758), rabbit anti-GAPDH (Cell Signaling, #2118L), and mouse anti-β-actin (Sigma-Aldrich, 8C-15). Each primary

antibody used has been validated for the relevant species and application by the manufacturer.

Annexin V staining

Staining of scr control and knockdown cells was performed using the APC Annexin V Apoptosis Detection Kit with 7-AAD (BioLegend, San Diego, CA) according to manufacturers` instructions. Stained cells were acquired on a BD FACS Calibur^{TM} flow cytometer (BD Biosciences, Heidelberg, Germany).

Cell synchronization and PI staining

For synchronization, cells were grown to 40% sub-confluency. Nocodazole (Sigma-Aldrich, Taufkirchen, Germany) was added to a final concentration of 50 ng/ml and cells were incubated for 20 hours before medium was changed to remove nocodazole and to release cells. Directly after nocodazole removal and 24- and 48- hours post-release cells were fixed with 70% ethanol, RNAse I-treated and stained with Propidium Iodide (Sigma-Aldrich, Taufkirchen, Germany) for 30 min at 4°C. Stained cells were acquired on a BD FACS Calibur^{TM} flow cytometer (BD Biosciences, Heidelberg, Germany).

Supplementary References

1 Nagy A, Lanczky A, Menyhart O, Gyorffy B. Validation of miRNA prognostic power in hepatocellular carcinoma using expression data of independent datasets. *Scientific reports* 2018; 8: 9227.

2 Comprehensive genomic characterization of squamous cell lung cancers. *Nature* 2012; 489: 519-525.

3 Comprehensive molecular profiling of lung adenocarcinoma. *Nature* 2014; 511: 543-550.

4 Schmidt LH, Spieker T, Koschmieder S, Schaffers S, Humberg J, Jungen D *et al*. The long noncoding MALAT-1 RNA indicates a poor prognosis in non-small cell lung cancer and induces migration and tumor growth. *Journal of thoracic oncology : official publication of the International Association for the Study of Lung Cancer* 2011; 6: 1984-1992.

5 Remmele W, Stegner HE. [Recommendation for uniform definition of an immunoreactive score (IRS) for immunohistochemical estrogen receptor detection (ER-ICA) in breast cancer tissue]. *Der Pathologe* 1987; 8: 138-140.

6 Eekels JJ, Pasternak AO, Schut AM, Geerts D, Jeeninga RE, Berkhout B. A competitive cell growth assay for the detection of subtle effects of gene transduction on cell proliferation. *Gene Ther* 2012; 19: 1058-1064.

7 Groß A, Chernyakov D, Gallwitz L, Bornkessel N, Edemir B. Deletion of Von Hippel-Lindau Interferes with Hyper Osmolality Induced Gene Expression and Induces an Unfavorable Gene Expression Pattern. *Cancers* 2020; 12.

8 Pfaffl MW. A new mathematical model for relative quantification in real-time RT-PCR. *Nucleic acids research* 2001; 29: e45.
