# Supplementary material for: NOP10 predicts lung cancer prognosis and its associated small nucleolar RNAs drive proliferation and migration
Source: Oncogene. 2020 Dec 7;40(5):909–21. doi: 10.1038/s41388-020-01570-y (PMC7862062; doi:10.1038/s41388-020-01570-y)
Supplement: Supplementary file 2 — Supplementary Tables [file 41388_2020_1570_MOESM2_ESM.pdf]

Supplementary Table 1. Baseline Characteristics of the Study Population (n= 172), related to Figure 1

**Parameter**	**Number of Patients (%)**
Age (years)	65 (34-83)
Sex	
male	142 (83%)
female	30 (17%)
smoker	
yes	133 (70.7 %)
no	39 (29.3 %)
Tumor histology	
adenocarcinoma	59 (34.3 %)
squamous carcinoma	81 (47.1%)
Large cell	25 (14.5 %)
other	7 (4.1 %)
pT	
pT1	62 (36.0 %)
pT2	91 (52.9 %)
pT3+pT4	19 (11.1 %)
pN	
pN0	106 (61.6 %)
pN1-2	66 (38.4 %)
pStage	
Not Available	4 (2.3 %)
pStage 1	131 (76.2 %)
pStage 2	17 (9.9 %)
pStage 3	18 (10.4 %)
pStage 4	2 (1.2 %)
Median Survival (months)	46.27 (±31 month)
NOP10 expression	
low	31 (18.0 %)
high	141 (82.0 %)

Supplementary Table 2: Multivariate analysis of factors associated with overall survival in NSCLC patients, related to Suppl. Figure 1I

**Risk Factor**	**HR (95% CI)**	**p-value**
Staging (II-IV/ I)	1.61 (1.09-2.38)	0.016
NOP10 protein (low/high)	0.35 (0.14- 0.92)	0.033
Sex (male/ female)	2.36 (1.04- 5.35)	0.039
Smoking (no/ yes)	0.65 (0.16- 1.38)	0.267
Age at diagnosis (≤60/>60)	1.0 (1.0- 1.0)	0.052
Histological Grade (low malignancy/ moderate+ high malignancy)	0.029 (0.001-1.623)	0.085

Supplementary Table 3. SnoRNAs enriched and depleted in tumor vs. normal tissue, related to Figure 3

snoRNAs marked in red simultaneously promote higher pseudouridylation of their target sites in tumor vs. matched normal (see Suppl. Table 3)

**snoRNA name**	**average (T)**	**average (N)**	**T/N**	**Log2FC(T/N)**
U18C	82,20848	21,76697	3,776753	1,917146
U78	491,1726	85,73821	5,728748	2,51822
U44	23720,85	12567,17	1,887524	0,916495
U74	1009,356	440,0391	2,293787	1,197731
mgU12-22/U4-8	11,30278	19,15246	0,590148	-0,76085
U14A	10,90798	4,464924	2,443039	1,288677
HBII-289	162,5246	459,6242	0,353603	-1,4998
HBI-6 (SNORA26)	6,373157	1,297656	4,911285	2,296101
HBII-13	226,289	390,7374	0,579133	-0,78803
U77	102,1291	10,84975	9,413036	3,23466
ACA9 (SNORA9)	13,36415	4,390575	3,043827	1,605886
U28	485,9898	122,5038	3,967141	1,9881
U75	496,3789	161,6574	3,070561	1,618502
U18B	545,7946	273,8575	1,992988	0,994933
U36B	117,6763	55,62151	2,115661	1,081109
U65 (SNORA65)	17,2263	5,928249	2,905799	1,538935
Z17B	119,457	35,84094	3,332977	1,736812
U52	3067,941	1638,204	1,872747	0,905156
U45C	1666,919	554,1474	3,008079	1,588842
snR38A	12,24837	2,395322	5,113452	2,354298

ACA51 (SNORA51)	10,30719	2,856425	3,608423	1,851369
HBI-100 (SCARNA3)	2,28297	0,60013	3,804128	1,927566
U67 (SNORA67)	7,430793	1,388266	5,352572	2,420232
HBII-240	32,06945	8,38463	3,82479	1,935381
HBII-180B	29,37224	8,024054	3,660524	1,87205
snR38C	288,8878	81,39701	3,54912	1,827461
ACA67B (SNORA80B)	17,38441	2,417725	7,190399	2,846072
U86	5,88211	1,139837	5,160483	2,367506
HBII-180A	192,3347	75,04979	2,562761	1,357699
U83B	530,3843	262,7696	2,018438	1,01324
HBII-95	20,86298	12,14347	1,718041	0,780764
U3-2	104,5189	18,17856	5,749568	2,523454
SNORA84	12,64188	1,389626	9,097327	3,185443
ACA42 (SNORA42)	8,794956	1,880927	4,675862	2,225232

Suppl. Table 4: Increase in pseudouridylation of respective snoRNA target sites as determined by Ψ-seq, related to Figure 3.

snoRNAs marked in red are enriched in tumor vs. matched normal (see Suppl. Table 2). Target rRNA positions for individual snoRNAs have been annotated based on Modomics [1]. SnOPY [2] annotations are given in column 3.

SnoRNA	Target RNA [1]	Target RNA [2]	Log2FC(T/N)	P Value
ACA36 (SNORA36A)	18S 105	18S 105	0,506507616	0,001191381
ACA36B (SNORA36B)	18S 105	18S 105	0,506507616	0,001191381
ACA50 (SNORA50)	18S 105	18S 105	0,506507616	0,001191381
ACA62 (SNORA76)	18S 105	18S 105	0,506507616	0,001191381
ACA9 (SNORA9)	28S 1662	28S 1670	0,607931071	0,003773914
ACA43 (SNORA43)	28S 4928	28S 4938	0,274478767	0,004214525
ACA42 (SNORA42)	18S 109	18S 109	0,32715463	0,005317231
ACA67 (SNORA80)	18S 109	18S 109	0,32715463	0,005317231
ACA67B (SNORA80B)	18S 109	18S 109	0,32715463	0,005317231
U66 (SNORA66)	18S 119	18S 119	0,266072956	0,01166257
ACA52 (SNORA52)	28S 1723	28S 1731	0,197505127	0,013975514
ACA31 (SNORA31)	18S 218<br>28S 3703	18S 218<br>28S 3713	0,1227333<br>0,4858697	0,037129618<br>0,04828447
ACA7 (SNORA7A)	28S 1771	28S 1779	0,150068195	0,038973871
ACA7B (SNORA7B)	28S 1771	28S 1779	0,150068195	0,038973871
U19-2 (SNORA74A (B))	28S 3731	28S 3741	0,258519692	0,040174303

Supplementary Table 5. Composition of the CRISPR/Cas9 KO Library (in separate Excel file), related to Figure 4

Supplementary Table 6. SnoRNAs enriched and depleted in A549 cells as identified by the CRISPR/Cas9 KO screen, related to Figure 4.

id	num	Neg. p-value	Neg. fdr	Neg. rank
SNORD14D	4	0.0040414	0.228342	8
SNORD76	3	0.011686	0.360726	14
**SNORA7A**	2	0.014621	0.388759	17
SNORA3_SNORA45A	6	0.025377	0.528128	20
SNORA5A	5	0.02553	0.528128	21
SNORD109A_SNORD109B	4	0.025705	0.528128	22
SNORD94	5	0.029736	0.560025	23
SNORD51	2	0.033241	0.591965	25
SNORD11B	3	0.035694	0.597543	27
SNORD67	4	0.050064	0.701499	30
SNORD7	5	0.060709	0.707667	38
SNORD43	2	0.064126	0.724629	40
SNORA59A	5	0.070304	0.727925	42
**SNORA65**	7	0.073195	0.727925	43
SNORD9	6	0.075254	0.727925	45
SNORD59A	2	0.078693	0.727925	48
SNORD58C	1	0.092362	0.750512	55
SNORD14C	3	0.094925	0.750512	56
SNORA70C	3	0.096305	0.750512	58
**SNORA7B**	1	0.1011	0.774543	59
**id**	**num**	**Pos. p-value**	**Pos. fdr**	**Pos. rank**
SNORA76	5	0.00040524	0.15099	1
SNORD86	7	0.0006681	0.15099	2
SNORA31	3	0.0026614	0.40099	3
SNORA63	4	0.0035595	0.402228	4
SNORA52	10	0.0045014	0.406931	5
SNORD50A	2	0.0087072	0.512101	7
SNORA71B	4	0.0099996	0.512101	8
SNORD88A	5	0.017272	0.624646	11
SNORD103A_SNORD103B	4	0.024216	0.624646	14
SNORA68	3	0.024501	0.624646	15
SNORA45B	2	0.024742	0.624646	16
SNORA13	4	0.029079	0.624646	18
SNORA80A	4	0.029473	0.624646	19
SNORA70E	4	0.033788	0.624646	21
SNORA79	6	0.034029	0.624646	22
SNORD68	3	0.034183	0.624646	23
SNORA73A	9	0.035168	0.624646	24
SNORA32	3	0.03703	0.624646	25
SNORD88B	3	0.0376	0.624646	26
SNORA19	2	0.038213	0.624646	27
SNORA26	1	0.038695	0.624646	28
SNORA77	5	0.050283	0.773833	29

SNORA71C	3	0.053021	0.773833	30
SNORA18	3	0.05324	0.773833	31
SNORA75	3	0.058037	0.773833	33
SNORD50B	3	0.067631	0.802361	37

Supplementary Table 7. Small guide RNA sequences used for knockout of individual snoRNAs and target genes

**target/ gRNA name**	**gRNA sequence**
SNORA7A7B-sgRNA-1-S	CACCGTCTGCCAGCTTCGGAAAGGG
SNORA7A7B-sgRNA-1-AS	AAACCCCTTTCCGAAGCTGGCAGAc
SNORA7A7B-sgRNA-2-S	CACCGTGACCTCCTGGGATCGCATC
SNORA7A7B-sgRNA-2-AS	AAACGATGCGATCCCAGGAGGTCAC
SNORA7A7B-sgRNA-3-S	CACCGTATTCTGCCAGCTTCGGAAA
SNORA7A7B-sgRNA-3-AS	AAACTTTCCGAAGCTGGCAGAATAc
SNORA7A7B-sgRNA-4-S	CACCGTCTCCAGATGCGATCCCAGG
SNORA7A7B-sgRNA-4-AS	AAACCCTGGGATCGCATCTGGAGAC
NOP10-sgRNA-1-S	CACCGCCAGTATTACCTCAACGAGC
NOP10-sgRNA-1-AS	AAACGCTCGTTGAGGTAATACTGGC
NOP10-sgRNA-2-S	CACCGAGATCGAGTCTATACGCTGA
NOP10-sgRNA-2-AS	AAACTCAGCGTATAGACTCGATCTC
NOP10-sgRNA-3-S	CACCGGAGCAGAAATTTGACCCGA
NOP10-sgRNA-3-AS	AAACTCGGGTCAAATTTCTGCTCC
NOP10-sgRNA-4-S	CACCGCCTGCTCAGCCCATCCTGCT
NOP10-sgRNA-4-AS	AAACAGCAGGATGGGCTGAGCAGGC
NOP10-sgRNA-5-S	CACCGCAACCGCGCCCTGTCCTCTG
NOP10-sgRNA-5-AS	AAACCAGAGGACAGGGCGCGGTTGC
NOP10-sgRNA-6-S	CACCGAGGACAGGGCGCGGTTGCT
NOP10-sgRNA-6-AS	AAACAGCAACCGCGCCCTGTCCTC
NOP10-sgRNA-7-S	CACCgTCATCTGGGGAGAACCGAGC
NOP10-sgRNA-7-AS	AAACGCTCGGTTCTCCCCAGATGAc
NOP10-sgRNA-8-S	CACCGACCCTCAGAGGACAGGGCG
NOP10-sgRNA-8-AS	AAACCGCCCTGTCCTCTGAGGGTC
SNORA65-sgRNA-1-S	CACCTGTTGGCTGGTGCAATCCAG
SNORA65-sgRNA-1-AS	AAACCTGGATTGCACCAGCCAACA
SNORA65-sgRNA-2-S	CACCAAACAGGGTTGTTCTTCATG
SNORA65-sgRNA-2-AS	AAACCATGAAGAACAACCCTGTTT

Supplementary Table 8. Sequences of primers used in PCR and quantitative RT-PCR

Primer name	Primer sequence
NOP10 (hum)-RT-AS	TACCTCAACGAGCAGGGAGA
NOP10 (hum)-RT-AS	CTGGGTCATGAGCACCTTGA
DKC1 (hum)-RT-S	CGGTCATCTCTACCTGCGAC
DKC1 (hum)-RT-AS	TGGCAGACTCACTGTAGTCAA
ACTIN (hum)-RT-S	AGGCACCAGGGCGTGAT
ACTIN (hum)-RT-AS	GCCCACATAGGAATCCTTCTGAC
SNORA7A/7B-genomic-S	GATTTGCTCAGCTGTTGGCATTC
SNORA7A/7B-genomic-S	ACGATCTTGGGCTTCACAAGG
NOP10-Genomic-1-S	GACGAACACGTGACGCGG
NOP10-Genomic-1-AS	CACGTATGACCTCACCCACT
NOP10-Genomic-2-S	TTTGCCCTTTTTCGCGCTG
NOP10-Genomic-2-AS	TCAATCGCCACGAGAGACTG
SNORA7A/B-RT-S	GTATTCTGCCAGCTTCGGAAAGG

References

1 Boccaletto P, Machnicka MA, Purta E, Piatkowski P, Baginski B, Wirecki TK *et al*. MODOMICS: a database of RNA modification pathways. 2017 update. *Nucleic acids research* 2018; 46: D303-d307.

2 Yoshihama M, Nakao A, Kenmochi N. snOPY: a small nucleolar RNA orthological gene database. *BMC research notes* 2013; 6: 426.
